# The Dynamic Interaction between Oil Palm and *Phytophthora palmivora* in Bud Rot Disease: Insights from Transcriptomic Analysis and Network Modelling

**DOI:** 10.3390/jof10030164

**Published:** 2024-02-20

**Authors:** Mariandrea García-Gaona, David Botero-Rozo, Leonardo Araque, Hernán Mauricio Romero

**Affiliations:** 1 Biology and Breeding Research Program, Colombian Palm Oil Research Center, Cenipalma, Calle 98 No. 70–91, Piso 14, Bogotá 111121, Colombia; mgarcia@cenipalma.org (M.G.-G.); dbotero@cenipalma.org (D.B.-R.); laraque@cenipalma.org (L.A.); 2 Department of Biology, Universidad Nacional de Colombia, Bogotá 111321, Colombia

**Keywords:** *Elaeis guineensis*, co-expression networks, effectors, gene expression profiles

## Abstract

Bud Rot, caused by *Phytophthora palmivora*, is considered one of the main diseases affecting African oil palm (*Elaeis guineensis*). In this study, we investigated the in vitro molecular dynamics of the pathogen–host interaction by analyzing gene expression profiles from oil palm genotypes that were either susceptible or resistant to the disease. We observed distinct interactions of *P. palmivora* with resistant and susceptible oil palms through co-expression network analysis. When interacting with susceptible genotypes, *P. palmivora* exhibited upregulation of carbohydrate and sulfate transport genes. These genes demonstrated co-expression with apoplastic and cytoplasmic effectors, including cell wall degrading enzymes, elicitins, and RxLR motif effectors. The pathogen manipulated susceptible oil palm materials, exacerbating the response and compromising the phenylpropanoid pathway, ultimately leading to susceptibility. In contrast, resistant materials exhibited control over their response through putative Heat Shock Proteins (HSP) that maintained homeostasis between primary metabolism and biotic defense. Co-expressed genes related to flavonoids, WRKY transcripts, lectin-type receptors, and LRR receptors may play important roles in pathogen control. Overall, the study provides new knowledge of the molecular mechanisms underlying the interaction between *E. guineensis* and *P. palmivora*, which can contribute to controlling Bud Rot in oil palms and gives new insights into the interactions of *P. palmivora* with their hosts.

## 1. Introduction

*Phytophthora palmivora* is a hemibiotrophic pathogen that causes diseases in various valuable tropical crops, including the African oil palm *Elaeis guineensis* [1]. Bud Rot (BR) caused by *P. palmivora* is considered one of the most devastating diseases affecting this crop [2]. However, many aspects of its host resistance mechanism and virulence remain unknown [3,4].

The molecular interactions between plants and microorganisms play an important role in determining microorganisms’ proliferation and disease potential [5]. Thus, a comprehensive understanding of these processes, including pathogen infection and plant defense, is essential for developing effective plant breeding strategies [6].

A previous study on the interaction between oil palm and *P. palmivora* showed that gene expression is altered when *P. palmivora* interacts with contrasting (susceptible and resistant) oil palm genotypes [4]. As a result of this analysis of differential gene expression, it was possible to identify the overexpression of genes associated with the salicylic acid (SA) pathway in resistant oil palm genotypes and few genes related to *P. palmivora* pathogenesis.

Although classical approaches for differential expression analysis are useful for identifying important genes related to a biological phenomenon, these approaches do not allow us to uncover relationships among genes by direct connection gene-to-gene based on their expression profiles. Co-expression analysis is one approach that allows for establishing those connections, depicts a holistic view of the plant–pathogen interaction, and finds candidate central genes related to metabolic and regulatory pathways [7]. By constructing gene co-expression networks and analyzing differential expression profiles, this study aimed to investigate the molecular differences underlying both compatible and incompatible interactions between *E. guineensis* and *P. palmivora*. By employing this approach, this work sought to advance our understanding of the mechanisms involved in the infection process while simultaneously enabling the identification of novel genes and the validation of candidate genes previously identified in related studies.

## 2. Materials and Methods

### 2.1. RNAseq Data

The RNAseq data used in this study were generated in a previous work, where contrasting materials were identified in their response to Bud Rot disease. In this study, the *E. guineensis* genotype Clon34 corresponds to a *P. palmivora* resistant genotype and Clon57 to a susceptible one [8]. Both were inoculated with 20 μL of *P. palmivora* at 300,000 zoospores/mL concentration. Leaf samples were collected, and total RNA was extracted at 24, 72, and 120 h post-inoculation (hpi) [4].

### 2.2. Analysis of Differentially Expressed Genes of P. palmivora and Oil Palm

Differentially expressed gene analyses were performed as described before [4]. Clean Illumina reads were mapped against the *Elaeis guineensis* genome [9]. The non-palm-mapped reads were subsequently mapped against the *P. palmivora* genome [10]. Differentially expressed genes in palm and pathogen organisms were obtained using the DESeq2 package [11]. Significant genes with a *p*-adjusted value ≤ 0.1 and an |Log 2-fold change| ≥ 2 were selected; controls correspond to palms without pathogen inoculation for plant comparisons, and *P. palmivora* petri dishes cultured for pathogen comparisons.

### 2.3. Annotation of Differentially Expressed Genes (DEGs) of P. palmivora and Oil Palm and Bioinformatic Identification of Effectors

DEGs of *P. palmivora* were annotated by Blast2GO_Basic 6 [12] and Pfam_33.1 [13]. Genes associated with cytoplasmic or apoplastic effectors were identified with the SecretSanta package V1, EffectorP_3.0 [14], and ApoplastP_1.0 [15]. For EffectorP and ApoplastP, localization of the genes was imputed if the probability was greater than 80%.

### 2.4. Construction of Co-Expression Networks of P. palmivora and Oil Palm

The differential expressed genes (DEGs) of resistant and susceptible interactions at each stage obtained above were used to construct co-expression networks for both the plant and pathogen. The igraph R package_1.2.8 was then used to construct the gene pair distance correlation matrix based on normalized read counts by variance stabilized in DESeq2_1.28.1; only sample columns of the corresponding phenomena network were included. The detailed process was as follows: first, the differentially expressed genes for each contrast (control vs. treatments) were joined in one list. After normalization of the counts, the matrix’s rows were filtered to include only differentially expressed genes, while the columns were filtered to include only the treatments. The resulting matrix had rows equal to DEGs and columns equal to treatments. Based on this, a network was constructed for each treatment. Second, a graph of adjacency based on Pearson’s pairwise correlation distances among genes was created. Third, the adjacency object was simplified (remove.multiple = TRUE, remove.loops = TRUE). Fourth, the edge weights were converted to absolute values. Fifth, edges below an absolute Pearson’s correlation coefficient of 0.8 were removed. Sixth, the remaining vertices that have no edges were removed. Seventh, the vertices were size-scaled to be proportional to the expression level of each gene represented by each vertex and multiplied by a factor of ten. Eighth, the edges’ widths were amplified by a factor of two. Based on Prim’s algorithm, the resulting graph adjacency object was converted into a minimum spanning tree and plotting.

Modules or communities of correlated genes within each network were identified using the “edge betweenness” function [16]. This function uses the number of shortest paths through a node (edge betweenness score) to rank the most important nodes that connect modules in the network. The iterative remotion of the highest-scored node will disconnect the modules and allow the construction of a hierarchical map of the graph. The parameters used for the function were weights NULL, directed = FALSE, merges = TRUE, bridges = TRUE, modularity = TRUE, and membership = TRUE. HUBs are nodes with many connections and are defined as “the principal eigenvector of AA^T^ where A is the graph’s adjacency matrix”. They were calculated according to Kleinberg’s [17] algorithm score metric; the parameters of the functions were scale = TRUE and weights = NULL. The vertex size was calculated using the HUB score to highlight the HUB nodes in the plots. Additional metrics such as density and diameter [18], weighted diameter, centralization degree, centralization closeness, centralization betweenness [19], and average path length [20] also helped to evaluate and compare the overall performance characteristics of these networks.

### 2.5. Gene Validation by qRT-PCR

Once *P. palmivora* and oil palm genes were identified, some were selected for qRT-PCR validation according to bibliographic references, and their possible importance was determined by gene co-expression analysis. The primers for each gene were designed with the CDS sequence of each gene in Primer-Blast tool of NCBI, with a Tm(min): 58 or 60 °C, Tm(opt): 60 or 62 °C, Tm(max): 63 or 65 °C (Table S3).

We used cDNA samples obtained from the oil palm *P. palmivora* inoculation experiment of [4] to validate genes. qRT-PCR reactions were performed with a volume of 10 μL, consisting of 6 μL of EVAGREEN qPCR master mix and 4 μL of cDNA of the sample, water, or cDNA of a pure culture of *P. palmivora* under the following amplification conditions: 95 °C/5 min, 95 °C/5 s, 60 °C/30 s and 72 °C/40 s for 42 cycles.

For *P. palmivora*, *EF1a* was used as a reference gene. This gene has been used to quantify the biomass of some oomycetes in differential expression experiments and to evaluate the relative expression of effector-associated genes [21,22,23,24]. To quantify the relative expression for the selected reference genes, we used the 2^−ΔΔct^ method [25].

## 3. Results

### 3.1. Oil Palm and Phytophthora Palmivora Transcriptome Analysis

**Figure 1:**
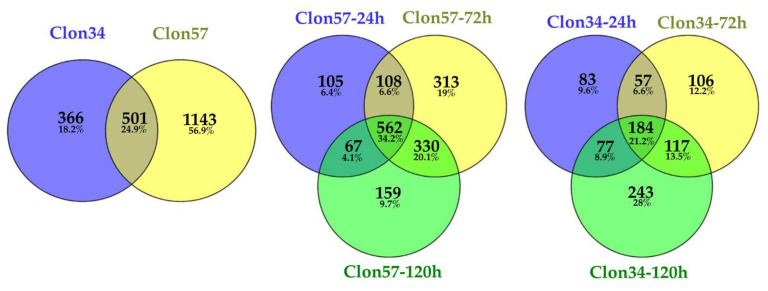
DEG Venn diagram of oil palm inoculated with *P. palmivora*. DEGs of susceptible (Clon57) and resistant (Clon34) *Elaeis guineensis* genotypes were analyzed at 24, 72, and 120 h post-inoculation.

To understand the *E. guineensis*–*P. palmivora* interactions, we conducted a transcriptional analysis of *P. palmivora* in vitro inoculated Clon34 and Clon57. A total of 2010 Differentially Expressed Genes (DEGs) were identified in response to *P. palmivora*. In total, 401, 464, and 621 genes were differentially expressed in resistant oil palm Clon34 at 24, 72, and 120 hpi, respectively. In contrast, in the susceptible oil palm Clone-57, 843, 1313, and 1118 genes were differentially expressed at different time points (Figure 1).

**Figure 2:**
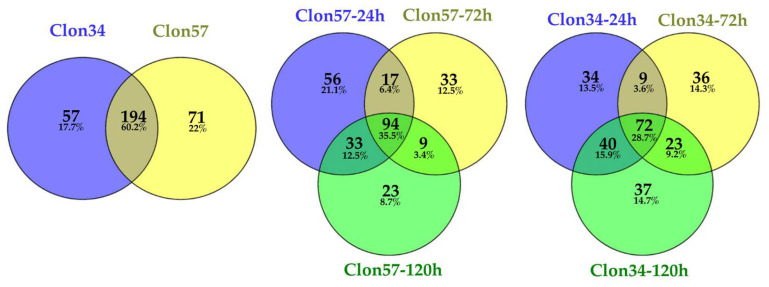
DEG Venn diagram of *P. palmivora* after inoculation on oil palm. DEGs of *P. palmivora* during the interaction with susceptible (Clon57) and resistant (Clon34) *Elaeis guineensis* genotypes were analyzed at 24, 72, and 120 h post-inoculation.

A total of 322 DEGs were identified in *P. palmivora* during the interaction with the resistant (Clon34) and susceptible (Clon57) oil palm genotypes. As shown in the Venn diagram (Figure 2), sets of genes are expressed at different time points and by the type of oil palm genotype. The comparison of the significant genes expressed by *P. palmivora* from each cultivar shows 57 genes unique to Clon34 and 71 genes exclusive to Clon57.

### 3.2. Bioinformatic Identification of P. palmivora Effectors

From significant DEGs, Secret Santa, EffectorP and ApoplastP were used to identify candidate effectors of *P. palmivora*, and Blast2GO and PFAM were used to annotate them. Table 1 shows a summary of the number of genes associated with selected categories of effectors. Secret Santa identified 85 secreted effectors; those proteins are secreted by the classical pathway [26]. According to the ApoplastP algorithm, 32 out of 322 are apoplastic effectors, with a probability greater than 80%, and those proteins accumulate between the space of the pathogens and plant cells. Among the apoplastic effectors identified, there were elicitins, hydrolases, Kazal proteinases (cysteine proteinases), and NPP1 proteins (necrosis and ethylene-inducing protein). EffectorP 3.0 identified 30 out of 322 as cytoplasmatic effectors; these included coding genes with RxLR motives, phosphate dikinases, hypothetical proteins, and peroxiredoxins. It is worth noting that 7 genes predicted as apoplastic effectors and 22 identified as cytoplasmatic were not identified as secreted by Secret Santa. This may suggest that those effectors are secreted via unconventional protein secretion pathways [27,28].

### 3.3. GO Enrichment of P. palmivora Genes in the Interaction with Clon34 and Clon57

To discern dissimilarities between the DEG pathways regulated in *P. palmivora* when interacting with susceptible and resistant oil palm materials, a functional enrichment analysis of Gene Ontology (GO) using Fisher’s exact test application of OmicsBox 3.0.29 software was conducted [29]. The DEGs at 24 h post-infection (hpi) were analyzed, corresponding to the biotrophic stage of *P. palmivora* during oil palm infection. The DEGs at 72 hpi and 120 hpi were grouped and analyzed separately, representing the necrotrophic stage of *P. palmivora* [1,4].

**Figure 3:**
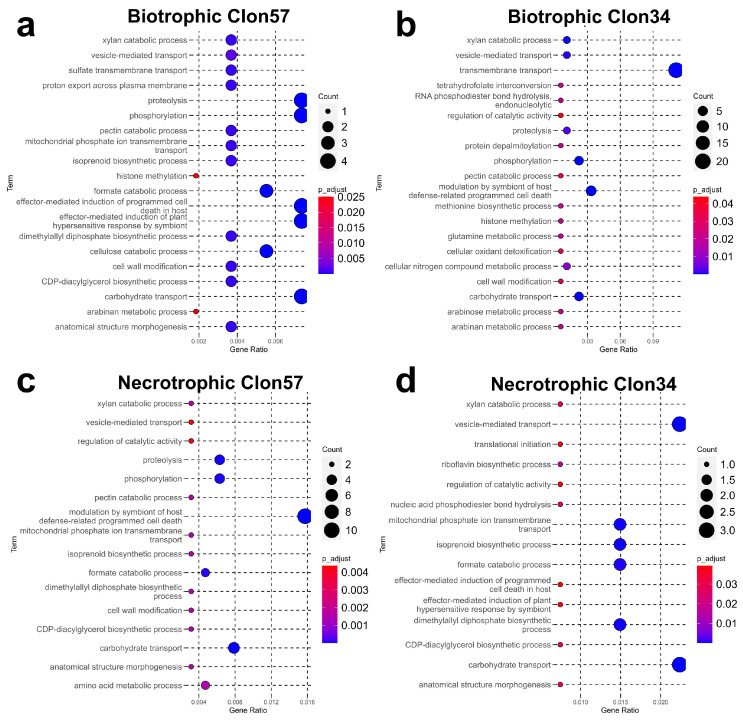
Dot plot of *P. palmivora* GO (Gene Ontology) term enrichment showing the top 20 biological process enriched pathways at the biotrophic (**a**,**b**) and necrotrophic (**c**,**d**) stage, in interaction with susceptible Clon57 (**a**,**c**) and resistant Clon34 (**b**,**d**). Each dot on the graph represents a biological process. The size of the dots represents the number of DEGs; the color of the dots represents the p-adjusted value from Fisher’s exact test. The *y*-axis lists the names of the biological processes while the *x*-axis displays the Gene Ratio, which is the proportion of the number of DEGs in the given pathway.

The Gene Ontology (GO) term for Biological Process associated with the biotrophic stage (Figure 3a,b) reveals that among the top 20 terms in the Clon57, there are categories related to biological processes such as effector-mediated induction of plant hypersensitive responses by symbionts, cellulose catabolic processes, carbohydrates, and sulfate transport that are significantly enriched in Clon57 but not in Clon34. At the biotrophic stage in Clon34, genes of *P. palmivora* are enriched in terms such as modulation by symbionts of host defense-related programmed cell death, glutamine metabolic processes, and cellular detoxification processes.

At the necrotrophic stage of *P. palmivora* interacting with Clon57, the DEGs are enriched in proteolysis, phosphorylation, amino acid metabolic processes, and modulation by symbionts of host defense-related programmed cell death (Figure 3c,d). In contrast, in Clon34, there is an enrichment of the vesicle-mediated transport and genes related to effectors that may be a late response to tissue infection.

These results imply that *P. palmivora* interacting with a susceptible oil palm material at the biotrophic stage can regulate effectors that modulate plant defense response. Also, there are enriched genes that have molecular functions altering the redox state of the plant and cellulase activities that contribute to invading the plant tissue.

### 3.4. Gene Co-Expression Network Analysis of P. palmivora

A co-expression network analysis was conducted to cluster genes with similar expression profiles to investigate the differences in the interaction of *P. palmivora* with susceptible and resistant oil palm genotypes. The analysis resulted in the construction of two separate gene co-expression networks (GCNs) based on the significant DEGs in *P. palmivora*. One GCN was derived from the interaction with the susceptible Clon57, while the other was derived from the resistant Clon34. Network construction was based on correlated gene pairs with a coefficient greater than 0.8.

**Figure 4:**
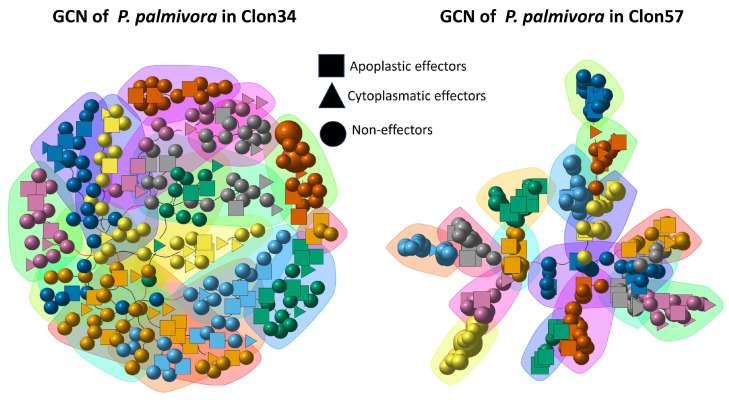
Gene co-expression networks (GCN) of *P. palmivora* inoculated in the resistant Clon34 and the susceptible Clon57. In the network, nodes represent differentially expressed genes, with the size of the nodes indicating their hub score. Triangular nodes represent cytoplasmic effectors, squares represent apoplastic effectors, and spheres represent non-effectors. The background colors correspond to modules.

The largest GCN constructed from the *P. palmivora*–Clon34 interaction grouped 251 genes out of the 322 DEGs, which formed 17 modules. Similarly, the largest GCN derived from the *P. palmivora*–Clon57 interaction grouped 265 genes out of 322 with 16 modules. Although both GCNs contained a similar gene content, the network topology exhibited distinct behaviors in response to Clon34 and Clon57 (Figure 4). The apoplastic and cytoplasmic secreted proteins were distributed throughout all modules in both GCNs (Figure S1). The highly connected genes, also known as hubs, were examined in both GCNs, which were chosen with a hub score threshold greater than 0.1. For the CGN Clon57, the hubs mainly belong to modules 2 and 16, while for the CGN Clon34, they belong to module 4 (Table S1).

Other observations about modularity revealed that in the network of Clon57, modules 5, 6, 8, and 12 exhibit an overrepresentation of genes expressed during the biotrophic stage, whereas modules 2, 3, and 9 are abundant in genes expressed during the necrotrophic stage. These modules contain genes belonging to hydrolase activities, oxidoreductase, or proteolysis. In the network of Clon34, modules 1, 2, 10, and 11 are abundant with genes exhibiting differential expression at the biotrophic stage (24 hpi) while modules 4, 8, and 14 show a high number of DEGs during the necrotrophic stage (72 and 120 hpi).

**Figure 5:**
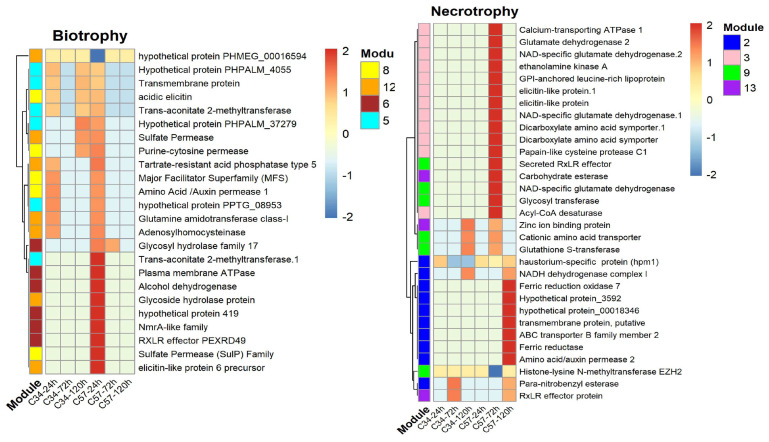
Heatmap depicting gene expression patterns of *P. palmivora* during its interaction with Clon57. Each heatmap highlights selected modules from the GCN *P. palmivora*–Clon57 interaction. Modules 5, 6, 8, and 12 are displayed in the biotrophic phase, while modules 2, 3, 9, and 13 are shown in the necrotrophic phase. The color scale indicates relative expression levels, with red representing higher expression and blue representing lower expression. Row clustering was performed using correlation analysis. Row names correspond to gene annotations, and column names indicate the three-time points (24, 72, and 120 hp) for Clon34 and Clon57.

The overall expression trends of these modules were visualized in heatmaps, which showed that gene expression is variable depending on the genotype. Specifically, during the biotrophic phase of the interaction with Clon57, *P. palmivora* exhibited upregulation of pathogenic-associated genes. For example, in module 6 (Figure 5), there is the transcriptional regulator NmrA-like family, which has been proven to have an important role in regulating the infection process at biotrophic stages in *P. capsici* [30], and it is co-expressed with the hmp1 (haustorial membrane protein 1) gene [31]. Our analysis found that hmp1 is also upregulated at 24, 72, and 120 hpi and was grouped into module 2. Together with NmrA, an RxLR effector (RXLR_40906), the glycosyl hydrolase family 17 (GH17) protein, an alcohol dehydrogenase, a plasma membrane ATPase, a hypothetical protein, and sugar transporter were co-expressed (Figure 5). *P. palmivora* did not express these genes during its interaction with Clon34, which was confirmed by validation of some of the genes through qRT-PCR (Figure S2). This implies that successful *P. palmivora* infection involves the early expression of the transcriptional factor NmrA-like and its co-expressed genes.

The other interesting modules during the biotrophic phase were modules 5, 8, and 12, which included several genes coding for amino acid transporters and sulfur permeases. These types of genes were co-expressed with genes associated with glucoside hydrolase proteins and elicitin effectors.

In Clon57, during the necrotrophic phase, modules 3, 9, and 13 were primarily expressed at 72 hpi. These modules contained genes associated with glutamate metabolism that were co-expressed with pathogen effectors, including elicitins, hydrolytic enzymes, and RxLR-motif effectors. In module 2, the gene *hpm1*, previously identified as a key virulence factor involved in the formation of haustorium membranes in other *Phytophthora* species [32], was found to be co-expressed with several genes encoding membrane transporters and permeases, which were predominantly upregulated at 120 hpi. This suggests that at this stage, *P. palmivora* had successfully established haustoria within the host plant tissue and that its nutrient uptake machinery was operational.

**Figure 6:**
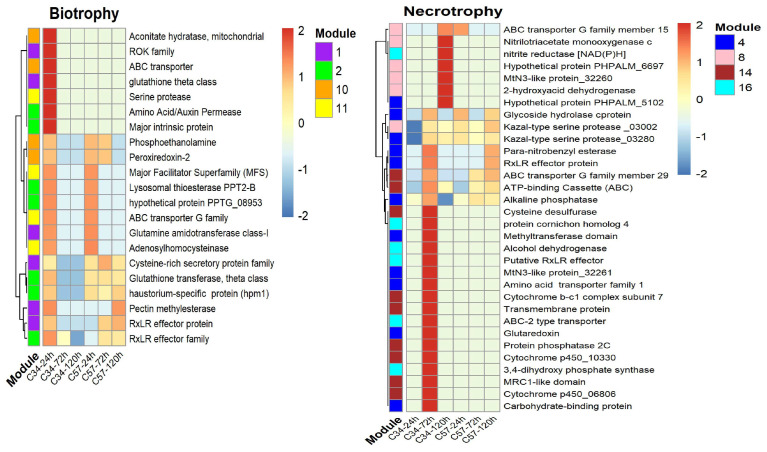
Heatmap depicting gene expression patterns of *P. palmivora* during its interaction with Clon34. Each heatmap highlights selected modules from the GCN *P. palmivora*–Clon34 interaction. Modules 1, 2, 10, and 11 are displayed in the biotrophic phase, while in the necrotrophic phase, modules 4, 8, and 12 are shown. The color scale indicates relative expression levels, with red representing higher expression and blue representing lower expression. Row clustering was performed using correlation analysis. Row names correspond to gene annotations, and column names indicate the three time points (24, 72, and 120 hpi) for Clon34 and Clon57.

In contrast, *P. palmivora* in the resistant Clon34 at the biotrophic stage upregulated proteases, a major intrinsic protein, ABC transporters, and a glutathione theta class protein. At the necrotrophic stage, the upregulated genes corresponded essentially to genes associated with the plasmatic membrane, such as ABC transporters and MtN3-like proteins. Other co-expressed proteins included alcohol dehydrogenase, glutaredoxin, and a putative RxLR effector. It seems that *P. palmivora* in Clon34 delays its infection process and up-regulates genes associated with detoxification (Figure 6). This expression pattern might reflect that the resistant oil palm genotype Clon34 alters the infection metabolism process of *P. palmivora*.

### 3.5. Gene Co-Expression Networks and Gene Ontology Analysis of Oil Palm Genotypes

**Figure 7:**
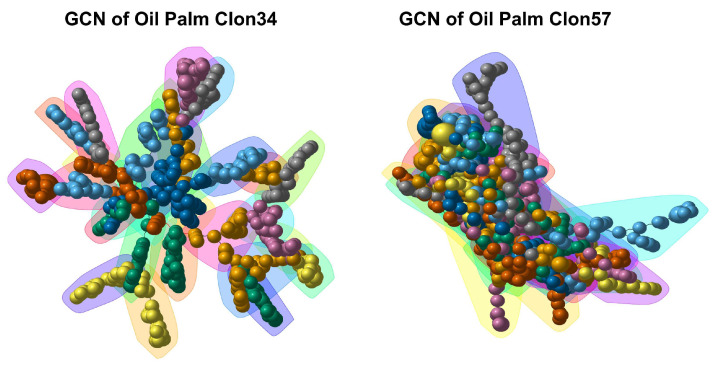
Gene co-expression network (GCN) of the oil palm resistant Clon34 and susceptible Clon57 during the interaction with *P. palmivora*. Nodes in the network represent significant differentially expressed (DE) genes, with the size of the nodes indicating their hub score. Background colors represent modules.

To obtain a more comprehensive understanding of the interplay between oil palm and *Phytophthora palmivora*, we conducted a co-expression analysis of the two oil palm genotypes (Clon34 and Clon57) during their interaction with *P. palmivora*. The topology of the resulting networks revealed differences in the plant defense response between the two plants. The largest gene co-expression network for Clon34 grouped 649 genes out of 1486 DEGs, with a correlation coefficient greater than 0.8, distributed in 27 modules, while GCN in Clon57 grouped 1327 DEGs, distributed in 35 modules (Figure 7).

**Figure 8:**
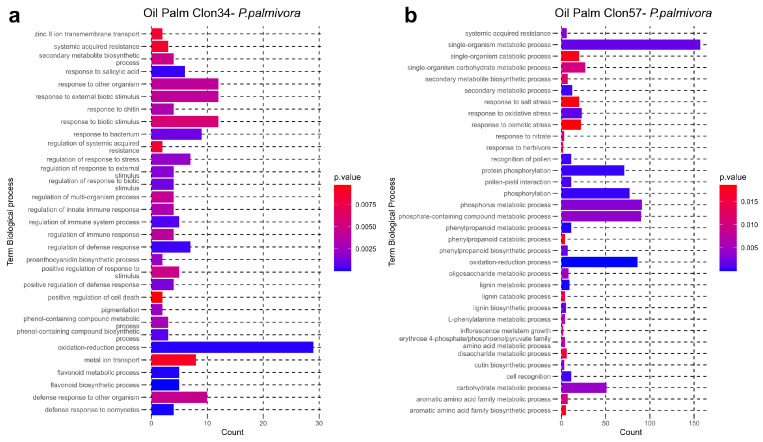
Bar graph of GO (Gene Ontology) term enrichment analysis of oil palm in response to *P. palmivora* infection of (**a**) Clon34 and (**b**) Clon57. Each graph shows the top 30 Biological Process significantly enriched terms (*p*-value < 0.05). The significance values are indicated by the color of the bars, while the bar height corresponds to the count of DEGs for each term.

To further investigate the functional properties of the genes in each network, we performed a Gene Ontology (GO) enrichment analysis using the GO enrichment tool of PlantRegMap with a threshold *p*-value < 0.05 and *Elaeis guineensis* as a background genome [33]. The two networks showed distinct gene ontology enrichment categories. In the case of the enrichment from network Clon34, in the top categories based on the p-value, we observed relevant categories included in biological processes such as the flavonoid biosynthetic process, response to biotic stimulus, defense response, response to oomycetes, response to salicylic acid and positive regulation of cell death (Figure 8a). Conversely, the network of Clon57 showed enrichment in categories related to protein phosphorylation, the oxide-reduction process, lignin metabolic process, phenylpropanoid metabolic process, and several responses associated with abiotic stress (Figure 8b). This analysis implies that the resistant Clon34 can control the infection by activating defense system pathways and utilizing salicylic acid (SA), flavonoids, and positive regulation of the cell death pathway. While susceptible Clon57 modulates pathways involved in plant defense, such as the phenylpropanoid or lignin metabolic process, and does not exhibit any enrichment in pathways of acid salicylic or positive regulation of cell death.

### 3.6. Central Genes and Modularity of Co-Expression Networks in Oil Palm

**Figure 9:**
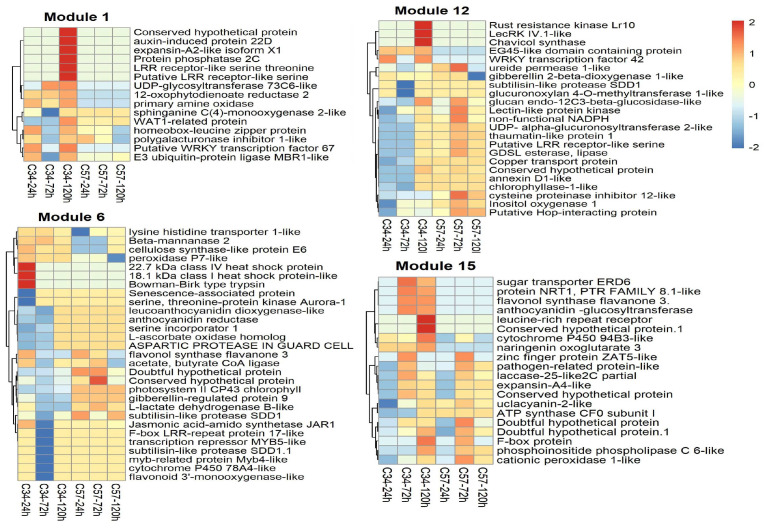
Heatmap depicting resistant oil palm Clon34 gene expression patterns during its interaction with *P. palmivora*. Each heatmap represents selected modules from the GCNof the oil palm Clon34 that reflect possible resistant pathways. The color scale indicates relative expression levels, with red representing higher expression and blue representing lower expression. Row clustering was performed using correlation analysis. Row names correspond to gene annotations, and column names indicate the three-time points (24, 72, and 120 hp) for Clon34 and Clon57.

Central or hub genes differ between the two networks. The primary central genes of the overall GCN of Clon34 belong to modules 6 and 24 (Table S2). Some GO terms identified for these modules are associated with photosynthesis and show downregulation at 24 and 72 hpi (Figure 9). This suggests that during the infection process under in vitro conditions, Clon34 reduces photosynthesis activity. For example, the gene coding for the photosystem I P700 apoprotein A1 is downregulated. This is not surprising, as it has been extensively reported that plants downregulate some of their primary metabolism to save energy and divert it to the defense response [34,35]. Furthermore, genes encoding a subtilisin-like SDD1, potentially associated with stomatal development, are also downregulated [36]. In module 6, some genes exhibited early upregulation at 24 hpi, including the two genes associated with the Heat Shock Protein-like (HSP), the jasmonic acid-amid synthetase JAR1, and the peroxidase P7-like, which are involved in early responses to oxidative stress due to mechanical damage of cuticles or cell walls [37,38,39].

**Figure 10:**
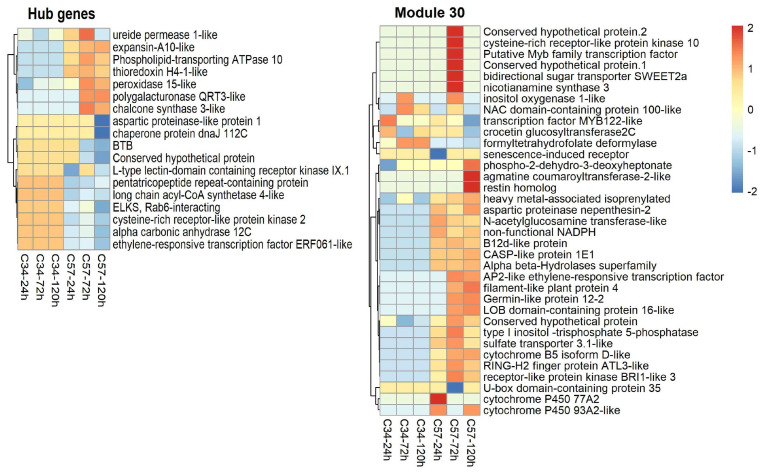
Heatmap depicting gene expression patterns of susceptible oil palm Clon57 during its interaction with *P. palmivora*. Each heatmap represents selected modules from the GCN oil palm-Clon57 that reflect its susceptible state. The color scale indicates relative expression levels, with red representing higher expression and blue representing lower expression. Row clustering was performed using correlation analysis. Row names correspond to gene annotations, and column names indicate the three time points (24, 72, and 120 hp) for Clon34 and Clon57.

Hub genes in susceptible Clon57 (modules 14 and 17) may reflect that the plant activates its defense metabolism to counteract the oxidative stress caused by the pathogen (Figure 10). For example, there is upregulation of chalcone synthase and the secreted peroxidase 15-like, which are involved in phenylpropanoid metabolism and oxidative stress. However, it also reflects some of the manipulating effects of the pathogen. For example, gene downregulation is related to the gene-encoded pentatricopeptide repeat-containing protein, which cleaves and matures mRNA in the chloroplast and other organelles [40]. Additionally, the transcription factor ERF061-like responsive to ethylene is downregulated, as well as the L-type lectin-domain IX.1-like and cysteine-rich receptor-like protein kinase 2, which have been linked to the PTI (pattern-triggered immunity) and callose deposition, respectively, in Arabidopsis [41,42]. Also, there is up-regulation of an expansin-A10-like, which may be induced by the pathogen to favor the loosening of the plant cell wall [43]. This process is regulated by the modulation of hormones like ABA and auxins, so there is also the co-expression of a tyrosine-sulfated glycopeptide receptor 1 in this module. A homolog of this gene in Arabidopsis has a suppressed salicylic acid dependent-response and enhanced susceptibility to biotrophic pathogens [44].

In Clon34, the central genes may indicate the critical pathways that are activated or suppressed to avoid the infection. Conversely, the hub genes identified in susceptible Clon57 indicate a dynamic response involving the upregulation of genes associated with oxidative stress, phenylpropanoid metabolism, and cell wall modification. The downregulation of genes related to chloroplast function and immunity pathways further suggests the limited capacity of Clon57 to suppress infection, either due to its genetic background or the manipulation of host processes by the pathogen.

**Figure 11:**
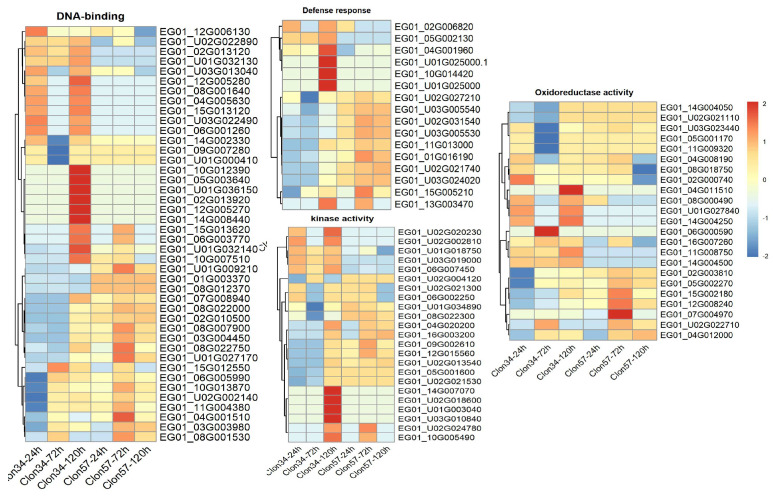
Heatmap depicting gene expression patterns of oil palm during its interaction with *P. palmivora*. Each heatmap represents the selected GO category Biological Process. The color scale indicates relative expression levels, with red representing higher expression and blue representing lower expression. Row clustering was performed using correlation analysis. Row names correspond to Gene ID; column names indicate the three time points (24, 72, and 120 hpi) for Clon34 and Clon57.

To investigate these critical pathways further, we selected GO categories, such as defense responses, response to oomycetes, oxidoreduction processes, DNA binding, and kinase activity. These categories indicate that certain genes related to the response to oomycetes and plant defense response are exclusively upregulated at late stages in the resistant Clon34. Furthermore, there are noticeable differences in the patterns of several genes associated with oxidation–reduction and DNA binding between the two materials. The activity of kinase proteins is another crucial factor in understanding plant changes during interactions with stress. Several unique genes in this category are either up- or down-regulated in Clon34 (Figure 11).

Assuming that a group of co-expressed genes may suggest the potential linkage of biological function between the genes, we sought to gain more insights into the critical pathways utilized by Clon34 during the response defense process by identifying unique up-regulated genes and analyzing their co-expressed genes in their co-expression network. For example, at 120 hpi in Clon34, the L-lectin domain-containing receptor IV gene was up-regulated and belonged to module 12 (Figure 9). This type of lectin receptor is a transmembrane protein associated with recognizing pathogens in the defense response of Arabidopsis [45]. It was co-expressed with a thaumatin transcription factor WRKY42, which has been linked to plant senescence and defense mechanisms [46,47], as well as a homolog of the rust resistance kinase Lr10.

Furthermore, module 15 of the GCN Clon34 had overexpressed genes associated with a membrane receptor kinase homologous to the SOBIR receptor of Arabidopsis (50% similarity, data not shown), which might be involved in the recognition complex of pathogens and triggered the expression of genes associated with the synthesis of flavones, flavonols, and anthocyanins, as well as a sugar transporter (Figure 9). Similarly, module 1 had an overexpressed membrane receptor and unique overexpressed genes associated with plant defense, including an LRR receptor-like serine, co-expressed with another LRR receptor, the transcription factor WRKY62, the 12-oxophytodienoate reductase 2 involved in jasmonic acid biosynthesis, and UDP-glycosyltransferase 73, which has been reported to be involved in the glycosylation of exogenous components and plant secondary products [48] (Figure 9). Overall, the modularity of the Clon34 network represents distinct pathways that lead to the overexpression of genes associated with membrane receptors and the trigger expression of secondary metabolite products, such as flavonoids.

On the contrary, modularity in susceptible Clon57 reflects the manipulation by the pathogen. In this case, a search for co-expressed genes associated with a state of susceptibility was performed in addition to the central genes explained above. For example, different authors have proposed that pathogens can manipulate host proteins to export nutrients to the apoplast [49,50]. Therefore, a search was performed for genes related to nutrient transportation, such as sugar and sulfur transporters. Regarding those processes, the product of SWEET genes that transport sugar from the cytoplasm to the apoplast and the sulfate transporter are usual genes targeted by effectors that cause the increase in their expression [51,52]; the results indicate that both types of genes are upregulated in Clon57. For example, relevant genes in module 30 in network Clon57 include the bidirectional sugar transporter SWEET2a, which is co-expressed with a sulfate transporter 3.1-like isoform X2, and the inositol oxygenase 1-like, which is involved in inositol metabolism and implicated in abiotic responses. While those genes are upregulated, they affect downregulated genes like the senescence-induced receptor-like; its homologs in rice and Arabidopsis are implicated in hypersensible response (HR) during biotic stress [53,54] (Figure 10). These findings collectively suggest that *P. palmivora* alters important plant physiological pathways, leading to an abiotic response rather than a biotic one.

## 4. Discussion

Data obtained from a simultaneous transcriptomic analysis of the interaction between oil palm and *P. palmivora* were used to perform Gene Ontology enrichment and co-expression network analyses, providing new insights into plant–pathogen interactions. The GO enrichment analysis allowed us to establish a comprehensive understanding of the successful infection process of oil palm at the biotrophic and necrotrophic stages by *P. palmivora*, where the pathogen regulates genes associated with carbohydrate transport, sulfate transmembrane transport, cellulose catabolic processes, extracellular secretion of effectors, and formate metabolism processes. The co-expression networks showed that highly connected genes or hubs were linked to genes associated with oxidative processes, NADH dehydrogenase, sulfur and glutamate metabolism, and effectors like elicitins.

The expression profile trends of selected modules of the networks point to specific genes expressed together during a successful infection of *P. palmivora*. At the biotrophic stage, the marker gene NmrA-like was upregulated and co-expressed with an RxLR motif gene, an alcohol dehydrogenase, the Putative Glycosyl hydrolase family 17 protein, a hypothetical protein PHPALM_419, and two genes associated with sugar transporters. The NmrA-like gene has been characterized as a transcriptional regulator of nitrogen metabolism and oxidative stress response in various plant pathogens [30,55]. In *Phytophthora capsici*, NmrA was found to be co-expressed with other effectors during the shift of biotrophic to necrotrophic lifestyle, and its overexpression resulted in reduced host colonization and altered gene expression, highlighting its crucial role in the pathogen’s life cycle [30,31]. In the case of Putative Glycosyl hydrolase family 17, which is an apoplastic effector with 1,3-β-glucanase activity, orthologs in *Cladosporium fulvum* have been proposed to play a role in degrading cell walls for penetration of the tissue or for the acquisition of sugar molecules during tomato infection [56,57]. As expected, in our analysis, this group of genes is co-expressed with sugar transporters that facilitate the uptake of carbohydrates. This group of genes could be markers of the biotrophic stage during *P. palmivora* infection of oil palm.

It is worth noticing in our analysis the expression of some of the apoplastic (elicitins, CWDE, and proteases) and cytoplasmatic effectors (RxLR) upregulated at biotrophic stages by *P. palmivora* during oil palm infection, co-expressed with genes related to nutrients metabolism such as sulfur, sugar, or amino acid transporters. The role of sulfur during plant pathogen infection is still poorly understood in oomycetes; however, in the genome of *P. infestans*, an overrepresentation of sulfate permease genes compared to fungi has been found [58]. Also, it has been shown that *P. infestans* upregulated sulfate permeases during the infection of tomatoes, suggesting an important role in pathogenesis [59]. Furthermore, to support the hypothesis that *P. palmivora* uptakes sulfur, a search for genes associated with sulfur permeases in the DEGs of the plant was performed; the results indicate there are contrasting genes exclusively upregulated in the susceptible ortet (Clon57), suggesting the gene expression’s manipulation by the pathogen to increase sulfur availability in the apoplast.

On the contrary, DEGs of *P. palmivora* are enriched in amino acid metabolism at the necrotrophic stage. Genes such as Amino Acid/Auxin Permease (AAAP), glutamate dehydrogenase, and the type IV NAD(P)H: quinone oxidoreductase, which are important for the uptake of amino acids and the process of nitrogenic nutrients, are also co-expressed with effectors like elicitins, and several hypothetical proteins.

Pathways of the integrated signals between the source of availability nutrients and the expression of virulence functions still need to be fully understood in the oomycetes [60,61]. However, in some microorganisms, pathogenicity is associated with sulfur [62] or iron metabolism [63]. Mainly, pathogens can alter the distribution of nutrients and availability between plants or the interphase, consequently affecting nutrient homeostasis and impacting the defense metabolism of the plant. For instance, siderophores of the phytopathogen *Erwinia chrysanthermi* promote growth in Arabidopsis by mobilizing iron from roots. This can interfere with the interplay between SA and JA [64]. Additionally, *Pseudomonas syringe* increases the uptake of asparagine when infecting tomato, which affects the expression synthesis of SA in the host [65,66]. Thus, the co-expression of *P. palmivora* genes related to nutrient uptake and effectors is expected under the assumption that they work together to alter metabolism to gain nutrients while avoiding the plant’s defense responses.

By analyzing the susceptibility response in Clon57, it was observed that *P. palmivora* suppressed the expression of certain genes associated with salicylic acid, jasmonic acid, ethylene, and cell death. Instead, genes related to abiotic stress were enriched and had positive expression in Clon57. In some pathosystems, activating these pathways may result in enhanced susceptibility [67]. Research has shown that pathogens can exploit the complex hormone pathways to take advantage of various levels of the plant’s physiological processes [68,69]. For example, in Clon57, several genes were associated with regulating the phenylpropanoid process, which is linked to the synthesis of papillae that can contain callose, lignin, and various phenolic conjugates. In this case, *P. palmivora* could manipulate this process to alter the composition of papillae. In barley, it has been demonstrated that a susceptible variety infected by *Blumeria graminis* produces a different composition of papillae that is ineffective compared to the effective papillae that completely restrict the pathogen [70].

Overall, during oil palm infection, the *P. palmivora* strategy appears to target hormone crosstalk to induce an “imaginary” abiotic response that promotes phenylpropanoid biosynthesis, alters cell death response, and causes nutrient leakage in oil palm. Further investigation is needed to examine the functionality of the effectors identified in this study and establish the mechanisms *P. palmivora* uses in oil palm infection.

The co-expression networks of the resistant oil palm Clon34 showed that the hub genes of the network are involved in negative control mechanisms of the photosynthesis and attenuation of some genes related to gibberellins and flavonoids. This negative regulation could be a strategy of Clon34 to prioritize the response to the pathogen and avoid the over-synthesis of unnecessary proteins. This could be explained by the upregulation of two genes coding for Heat Shock Proteins (HSP) at the early stages of infection. These proteins are chaperones that regulate multiple functions in plants. Recent evidence shows these proteins are important for maintaining protein homeostasis during plant defense response [71].

Clon34 also has enriched genes related to flavonoid biosynthesis, plant defense response and response to oomycetes GO categories. Focusing on those types of genes, we analyzed individual groups of co-expressed genes or modules that showed upregulation associated with flavonoids, anthocyanins, pathogen-related proteins (PR1), thaumatin-like proteins, or L-type lectins receptors. Most of these types of genes were co-expressed with several genes associated with LRR receptor-like serine threonine-protein kinases, WRKY or MYB transcription factors, and genes involved in phenylpropanoid pathways. This set of enriched categories, besides the differential expression of Heat Shock Proteins, suggests that different pathways are activated through kinases and transcription factors, ending up in the expression of flavonoids, naringenins, and pathogen-related proteins and inhibiting the *P. palmivora* infection process. In many plants, flavonoids and naringenins are hallmarks of resistance to biotic stress, and some of them have antibiotic effects on fungi, bacteria, and oomycetes [72,73].

In summary, we hypothesize that the early detection of *P. palmivora* by Clon34 leads to a defense response induced by the hydrolysis of oil palm cell walls and cuticles, generating DAMs (Damage-Associated Molecular Patterns) or by the perception of PAMPs (Pathogen-Associated Molecular Patterns) or elicitors. These molecules may be recognized by lectin or LRR receptors, which activate complexes in the membrane and kinases, activating heat shock proteins that modulate the response of primary metabolism and promote biotic defense responses. The activation of different WRKY transcription factors initiates the biosynthesis of SA and jasmonic acid (JA), which regulate the phenylpropanoid pathway, promoting the synthesis of secondary metabolites such as flavonoids, anthocyanins, and naringenin. In addition, other receptors, such as lectin or LRR receptors, are upregulated to increase pathogen perception. However, it is still unclear which specific molecules initiate the plant defense response, as our analysis began at 24 hpi. Further investigation into the hours preceding this time point is necessary to understand the exact mechanisms of pathogen perception, as this may explain why Clon57 cannot initiate a similar response.

In conclusion, this work extends the number of interplaying genes during *P. palmivora* infection and *E. guineensis* defense, and complements the model proposed by others [4] of understanding the molecular interactions between oil palm and *P. palmivora*. Also, we look forward to confirming the function of the genes by functional characterization of the genes presented here in this study. Finally, we hope the insights presented here will improve disease control by improving crop breeding.

## Figures and Tables

**Table 1:** List of secretome classes of *P. palmivora* in interaction with oil palm.

Effector Category	Number of Proteins
**Signal peptide genes (Secret Santa)**	80
Apoplastic effectors (ApoplastP)	30
With signal peptide	23
Without signal peptide	7
**Citoplasmatic effectors (EffectorP 3.0)**	29
With signal peptide	7
Without signal peptide	22
RxLR effectors	13
**Possible Elicitors**	
Elicitines	16
Cellulose-binding domain, fungal	2
Carbohydrate binding proteins	8
NPP1 (necrosis and ethylene-inducing protein)	4
**Proteases**	
Serine protease	5
Cysteine protease	2
**Cell wall degrading enzymes (CWDE)**	
Glycosyl hydrolase	8
Endoglucanase	6
Pectinesterase	3
**Others**	
Cysteine-rich proteins	2
ABC transporters	12
Kinase	5
Oxidase	4
Hypothetical proteins	28

## Data Availability

Data are available upon request from the authors.

## Supplementary Materials

The following supporting information can be downloaded at: https://www.mdpi.com/article/10.3390/jof10030164/s1, Figure S1: Distribution of *P. palmivora* effectors in the different modules of the gene co-expression networks of the interaction with resistant Clon34 (A) and susceptible Clon57 (B). The plots show cytoplasmic (blue) and apoplastic (red) effectors; Figure S2: Bar graph of the relative expression of *P. palmivora* genes associated with effectors during the infection of Clon34 or Clon57. The scatterplot shows the association between the relative expression values obtained by RNAseq analysis and the qRT-PCR methodology. CBM: Cellulose binding domain, CBX: carboxylase, Eli: elicitin, HMP1: haustoria membrane protein 1, Nep1: necrosis ethylene protein 1, PPDK: pyruvate, phosphate dikinase 1; RXLR: RxLR effector motive. Table S1: Gene hub description of the co-expression network of *P. palmivora* in the interaction with Clon57 and Clon34; Table S2: Gene hub description of the co-expression network of oil palm Clon57 and Clon34 in the interaction with *P. palmivora*; Table S3: List of primers for qRT-PCR.
