# Supplementary material for: The Dynamic Interaction between Oil Palm and Phytophthora palmivora in Bud Rot Disease: Insights from Transcriptomic Analysis and Network Modelling
Source: J Fungi (Basel). 2024 Feb 20;10(3):164. doi: 10.3390/jof10030164 (PMC10971723; doi:10.3390/jof10030164)
Supplement: Supplementary file 1 [file jof-10-00164-s001.zip › Supp JOF/Table S2.pdf]

Table S2. Gene hubs description of the co-expression network of Oil palm Clon57 and Clon34

Gene	Module	Degree	Hub Score	Betweenness	Closeness	Annotation
EG01_06G000840	6	13	1	36849	0.00012915	serine incorporator 1
EG01_11G011640	24	11	0.64186	23656	0.00012028	photosystem I P700 apoprotein A1 (chloroplast)
EG01_U02G015490	6	5	0.31379	121540	0.00013854	Doubtful hypothetical protein
EG01_12G014890	6	4	0.27983	5766	0.00011945	flavonol synthase flavanone 3-hydroxylase-like isoform X3 PRUPE_ppa007719mg
EG01_07G001640	6	2	0.23512	647	0.00011922	cytochrome P450 78A4-like
EG01_U03G006380	6	2	0.23512	647	0.00011922	photosystem II CP43 chlorophyll apoprotein (chloroplast)
EG01_06G003190	2	3	0.22288	11969	0.00011207	Putative protein
EG01_06G012860	6	1	0.22019	0	0.00011919	subtilisin-like protease SDD1
EG01_08G004430	6	1	0.22019	0	0.00011919	mannan endo-1%2C4-beta-mannosidase 2-like
EG01_09G002150	6	1	0.22019	0	0.00011919	flavonoid 3'-monooxygenase-like
EG01_09G007280	6	1	0.22019	0	0.00011919	myb-related protein Myb4-like
EG01_10G005990	6	1	0.22019	0	0.00011919	L-ascorbate oxidase homolog
EG01_13G004860	6	1	0.22019	0	0.00011919	gibberellin-regulated protein 9 isoform X2
EG01_14G004050	6	1	0.22019	0	0.00011919	leucoanthocyanidin dioxygenase-like
EG01_U02G019480	6	1	0.22019	0	0.00011919	subtilisin-like protease SDD1
EG01_15G004150	24	2	0.19867	5120	0.00011179	vignain isoform X1
EG01_06G005990	24	2	0.19768	647	0.00011162	WRKY transcription factor 42 OS%3DArabidopsis thaliana GN%3DWRKY42 PE%3D2 SV%3D1
EG01_02G006090	24	1	0.18512	0	0.00011159	Protein CDI OS%3DArabidopsis thaliana GN%3DCDI PE%3D2 SV%3D1
EG01_05G001170	24	1	0.18512	0	0.00011159	naringenin%2C2-oxoglutarate 3-dioxygenase-like
EG01_08G008010	24	1	0.18512	0	0.00011159	dihydroflavonol-4-reductase-like
EG01_11G009320	24	1	0.18512	0	0.00011159	leucoanthocyanidin dioxygenase-like
EG01_U02G001520	24	1	0.18512	0	0.00011159	Doubtful hypothetical protein
EG01_U02G021110	24	1	0.18512	0	0.00011159	naringenin%2C2-oxoglutarate 3-dioxygenase-like
EG01_U03G019560	24	1	0.18512	0	0.00011159	Sugar isomerase domain-containing family protein
EG01_12G008030	14	5	0.12941	112199	0.000136	Conserved hypothetical protein
EG01_U02G028840	6	4	0.11468	105621	0.00013864	Senescence-associated family protein
EG01_12G011430	6	5	0.10945	3865	0.00011102	acetate, butyrate--CoA ligase AAE7%2C peroxisomal-like isoform X1
EG01_U01G000410	6	1	0.090501	0	0.00012715	transcription repressor MYB5-like
EG01_U02G013300	6	1	0.090501	0	0.00012715	cellulose synthase-like protein E6 isoform X2
EG01_04G007000	2	7	0.081827	10821	0.00010487	Putative: CAP-Gly domain-containing linker protein 1
EG01_15G003690	6	1	0.080707	0	0.00011088	lysine histidine transporter 1-like
EG01_U02G010540	6	1	0.080707	0	0.00011088	Conserved hypothetical protein
EG01_10G001710	6	1	0.067812	0	0.00011068	F-box LRR-repeat protein 17-like isoform X2 GSCOC_T00016147001
EG01_U02G014040	6	1	0.067812	0	0.00011068	protein ASPARTIC PROTEASE IN GUARD CELL 2-like
EG01_13G008880	2	1	0.049075	0	0.00010449	Doubtful hypothetical protein
EG01_U03G010640	24	2	0.046962	4487	0.00010441	ribulose bisophosphate carboxylase (chloroplast)
EG01_15G006050	24	1	0.043526	0	0.0001041	cation H(+) antiporter 19 Sb09g008200
EG01_01G007190	14	5	0.041066	89741	0.00013144	S-type anion channel SLAH4-like
EG01_12G009390	11	4	0.033322	121417	0.00013862	ATP synthase CF1 beta subunit (chloroplast)
EG01_U03G026590	14	3	0.032992	32276	0.00012671	Photosystem II protein D2 (Fragment)
EG01_08G007900	14	2	0.030427	647	0.00012503	WRKY transcription factor 42 OS%3DArabidopsis thaliana GN%3DWRKY42 PE%3D2 SV%3D1
EG01_13G004830	14	2	0.030427	647	0.00012503	OSJNBa0018M05.15 protein
EG01_04G001510	2	3	0.02718	5127	9.8357E-05	transcription factor MYB108-like
EG01_12G000830	2	3	0.027168	1937	9.8261E-05	7-deoxyloganetic acid glucosyltransferase-like
EG01_01G009510	6	2	0.025734	647	0.00010361	18.1 kDa class I heat shock protein-like
EG01_06G005050	6	2	0.025734	647	0.00010361	Bowman-Birk type trypsin inhibitor-like
EG01_01G002720	6	1	0.02525	0	0.00012723	serine, threonine-protein kinase Aurora-1
EG01_U02G005600	6	1	0.02525	0	0.00012723	anthocyanidin reductase
EG01_06G002510	6	1	0.0241	0	0.00010358	peroxidase P7-like
EG01_U02G013040	6	1	0.0241	0	0.00010358	22.7 kDa class IV heat shock protein-like
EG01_01G003130	2	1	0.0236	0	9.8203E-05	Conserved hypothetical protein
EG01_12G011390	2	1	0.0236	0	9.8203E-05	BRCT domain-containing protein At4g02110

EG01_13G006260	2	1	0.0236	0	9.8203E-05	1%2C4-alpha-glucan-branching enzyme%2C chloroplastic amyloplastic-like isoform X2 VIT_18s0001g00060
EG01_U03G010740	2	1	0.0236	0	9.8203E-05	rab9 effector protein with kelch motifs-like isoform X3
EG01_15G012720	3	6	0.018775	91310	0.00012695	transmembrane protein 56-B-like
EG01_02G009450	21	6	0.016657	115141	0.00013618	formin-like protein 5
EG01_U01G038210	14	4	0.014632	1938	0.00012123	transcription factor HEC2-like
EG01_12G010660	24	2	0.014613	3852	9.7914E-05	beta-glucosidase 22-like
EG01_01G012290	14	1	0.011844	0	0.00012114	protein EXORDIUM-like 2
EG01_06G011310	14	1	0.011844	0	0.00012114	Nucleotide pyrophosphatase phosphodiesterase (Fragments) OS%3DHordeum vulgare GN%3Dnpp PE%3D1 SV%3D2 MTR_4g103520
EG01_10G000690	11	2	0.010391	35807	0.00012922	protein SULFUR DEFICIENCY-INDUCED 1-like
EG01_16G001050	14	2	0.010212	29351	0.00011847	phosphoenolpyruvate carboxylase 4 isoform X1
EG01_13G004950	14	2	0.010211	1935	0.00011719	UDP-glycosyltransferase 73C5-like
EG01_11G002310	11	1	0.0096107	0	0.00012721	F-box protein At4g35930 isoform X1
EG01_12G005270	14	1	0.0087757	0	0.00011567	Putative WRKY transcription factor 70
EG01_U01G027840	14	1	0.0087757	0	0.00011567	zeaxanthin epoxidase%2C chloroplastic-like
EG01_U03G003900	5	9	0.0082207	35813	0.00012703	glutathione S-transferase U17-like
EG01_U02G025220	6	1	0.0074222	0	9.7097E-05	Jasmonic acid-amido synthetase JAR1 OS%3DOryza sativa subsp. japonica GN%3DGH3.5 PE%3D2 SV%3D1
EG01_U02G032940	6	1	0.0074222	0	9.7097E-05	L-lactate dehydrogenase B-like
EG01_08G007270	2	2	0.006425	3852	9.2575E-05	protein E6-like
EG01_U02G016040	2	2	0.0063877	647	9.2404E-05	polygalacturonase-like
EG01_12G010490	2	1	0.0059846	0	9.2473E-05	caffeoylshikimate esterase-like isoform X1
EG01_01G001170	2	1	0.005982	0	9.2387E-05	tryptophan aminotransferase-related protein 3-like
EG01_U03G009790	3	4	0.0051719	10803	0.00011779	12-oxophytodienoate reductase 1-like
EG01_04G013390	3	4	0.0051342	2581	0.00011743	inorganic phosphate transporter 1-4-like
EG01_11G012370	25	3	0.0048538	78725	0.00012192	2-oxoglutarate dehydrogenase%2C mitochondrial-like
EG01_09G010730	3	3	0.0047353	1293	0.00011737	Putative protein
EG01_U02G025480	21	4	0.0046414	86559	0.00013123	ent-kaur-16-ene synthase%2C chloroplastic-like
EG01_U01G040590	3	1	0.0041339	0	0.00011732	glutathione S-transferase U17-like
EG01_08G001640	21	2	0.0039857	5751	0.00012544	Putative WRKY transcription factor 50
EG01_U02G014110	21	2	0.0039164	647	0.00012519	Putative ovule protein
EG01_U02G031020	24	3	0.0037055	3219	9.2166E-05	Cytochrome c biogenesis CcmF N-terminal-like mitochondrial protein 2 (Fragment) OS%3DArabidopsis thaliana GN%3DCCMFN2 PE%3D1 SV%3D2
EG01_15G000990	21	1	0.0036677	0	0.00012516	protein jagged-1b-like
EG01_06G011230	14	1	0.0032217	0	0.00011241	Cytochrome c oxidase subunit 3 OS%3DGlycine max GN%3DCOX3 PE%3D2 SV%3D1
EG01_14G002090	14	1	0.0032217	0	0.00011241	short-chain dehydrogenase TIC 32%2C chloroplastic-like
EG01_U02G011520	14	1	0.0032217	0	0.00011241	tubulin beta chain-like
EG01_11G006370	5	4	0.0029857	4498	0.00011758	cytochrome P450 84A1-like
EG01_08G010750	9	3	0.0027843	20940	0.00011833	Putative serine
EG01_01G004010	11	3	0.0027065	35555	0.00012098	cationic amino acid transporter 5-like isoform X1
EG01_15G000370	5	2	0.0025591	3852	0.00011755	Putative expressed protein
EG01_15G004660	5	2	0.0025435	1292	0.00011744	cinnamyl alcohol dehydrogenase 2-like
EG01_03G001990	5	2	0.0025318	647	0.00011741	LOB domain-containing protein 1-like
EG01_10G006190	5	2	0.0025318	647	0.00011741	protein TRANSPARENT TESTA 12-like
EG01_08G007000	14	2	0.0024168	28800	0.00011121	gibberellin 2-beta-dioxygenase-like
EG01_12G005880	14	2	0.0024119	1292	0.00010898	glutathione transferase GST 23-like
EG01_01G010710	5	1	0.002371	0	0.00011738	Hypothetical protein
EG01_06G005360	5	1	0.002371	0	0.00011738	LOB domain-containing protein 25-like
EG01_U01G023890	2	2	0.0020001	3215	0.00008742	cytochrome P450 71A1-like
EG01_02G008610	2	1	0.0018423	0	8.7192E-05	monogalactosyldiacylglycerol synthase 1%2C chloroplastic isoform X1
EG01_10G012090	25	3	0.0017748	83300	0.00011719	acidic endochitinase-like
EG01_11G013000	3	2	0.0016211	5751	0.00010966	L-type lectin-domain containing receptor kinase IV.2-like
EG01_06G002380	3	2	0.0016014	3215	0.00010957	lachrymatory-factor synthase-like
EG01_08G022000	3	2	0.0015812	647	0.00010916	transcription factor RAX3-like
EG01_12G010090	21	3	0.0015424	84129	0.00012644	potassium transporter 5-like
EG01_U03G023440	21	3	0.001541	1937	0.00012105	1-aminocyclopropane-1-carboxylate oxidase-like

EG01_U01G019690	25	2	0.0014949	647	0.00011303	thaumatin-like protein 1b
EG01_U02G024780	3	1	0.0014917	0	0.00010945	receptor-like protein kinase 5
EG01_10G009070	3	1	0.0014808	0	0.00010913	RPM1-interacting protein 4 isoform X2
EG01_10G012530	3	1	0.0014808	0	0.00010913	endoglucanase 6
EG01_U02G010330	21	4	0.0014444	5140	0.00011624	Conserved hypothetical protein
EG01_01G001520	3	1	0.0013658	0	0.00010909	Doubtful hypothetical protein
EG01_14G004250	3	1	0.0013658	0	0.00010909	naringenin%2C2-oxoglutarate 3-dioxygenase-like
EG01_U01G008180	21	1	0.0013387	0	0.00012096	Pectinesterase
EG01_16G007260	24	2	0.0011469	1935	8.7025E-05	short-chain type dehydrogenase reductase-like DCAR_016868
EG01_07G004970	21	1	0.0011296	0	0.00011581	2-alkenal reductase (NADP(+)-dependent)-like
EG01_05G004720	24	1	0.0010687	0	8.6979E-05	Putative acyl-activating enzyme 5%2C peroxisomal
EG01_03G004450	11	4	0.00099702	32945	0.00011357	transcription factor MYB108-like
EG01_16G007280	11	3	0.00090375	2579	0.0001123	Expressed protein
EG01_U01G015290	5	4	0.00081648	2581	0.00010936	cytochrome P450 71A1-like
EG01_09G003700	9	4	0.00077825	19301	0.00011067	Conserved hypothetical protein
EG01_12G004120	26	2	0.00076372	28247	0.00010477	zinc transporter 8-like
EG01_U03G026750	25	8	0.00075405	54832	0.00011128	Conserved hypothetical protein
EG01_U02G020230	14	2	0.0007428	647	0.00010182	receptor-like protein kinase HSL1
EG01_08G015070	5	1	0.00065741	0	0.00010927	anthocyanidin 5%2C3-O-glucosyltransferase-like
EG01_U03G008040	5	1	0.00065741	0	0.00010927	ATP synthase subunit alpha
EG01_08G010840	9	2	0.00065463	647	0.00010994	Calmodulin-binding protein 60 D OS%3DArabidopsis thaliana GN%3DCBP60D PE%3D2 SV%3D1
EG01_02G003870	5	3	0.00065212	3221	0.00010936	E3 ubiquitin-protein ligase RGLG1-like
EG01_08G008420	5	2	0.00059801	647	0.00010917	scopoletin glucosyltransferase-like
EG01_U01G030100	5	1	0.00055746	0	0.00010912	Isoform 2 of Indole-3-acetic acid-amido synthetase GH3.2 OS%3DArabidopsis thaliana GN%3DGH3.2
EG01_U02G003400	5	1	0.00055746	0	0.00010912	polyphenol oxidase%2C chloroplastic-like
EG01_U02G003450	25	5	0.00054571	34976	0.00011035	extensin-like
EG01_13G010660	2	3	0.00050987	2579	8.2795E-05	short-chain dehydrogenase TIC 32%2C chloroplastic-like
EG01_U03G022860	3	4	0.00044884	5139	0.00010256	zingipain-2-like
EG01_U02G030500	3	2	0.00038041	2576	0.0001024	GABA transporter 1-like
EG01_U02G014880	21	2	0.00036684	83367	0.00012192	OSJNBa0010H02.17 protein
EG01_U01G036410	21	2	0.00036232	647	0.00011228	premnaspirodiene oxygenase-like
EG01_02G013940	3	1	0.00034816	0	0.00010196	Conserved hypothetical protein
EG01_13G004610	21	2	0.00034298	1935	0.00010818	mannose-specific lectin-like
EG01_15G007950	21	1	0.00033962	0	0.00011688	receptor-like cytosolic serine threonine-protein kinase RBK1 isoform X2 SETIT_006150mg
EG01_01G016190	21	2	0.00033959	647	0.00010813	pathogenesis-related protein PR-4-like
EG01_16G005670	21	2	0.00033959	647	0.00010813	senescence-specific cysteine protease SAG39-like
EG01_U02G006850	21	1	0.00033931	0	0.00011226	ent-kaur-16-ene synthase%2C chloroplastic-like
EG01_U02G020020	25	1	0.00032915	0	0.00010533	cytochrome P450 71A1-like
EG01_13G002140	25	4	0.00027324	3863	0.00010394	RING-H2 finger protein ATL16-like
EG01_12G006130	24	2	0.00027089	1292	8.2413E-05	transcription factor MYB122-like
EG01_U03G005540	25	4	0.0002701	2581	0.0001039	pathogenesis-related protein 1-like
EG01_01G011540	9	3	0.00026461	14435	0.00010377	Putative glucan 1%2C3-beta-glucosidase A
EG01_U03G018480	9	3	0.00026117	3221	0.00010338	Germin-like protein 8-5 OS%3DOryza sativa subsp. japonica GN%3DOs08g0189400 PE%3D2 SV%3D1
EG01_U01G013780	20	3	0.00025729	12011	0.00010622	Conserved hypothetical protein
EG01_04G015290	4	3	0.00025596	16260	0.00010638	anthocyanidin 3-O-glucosyltransferase 2-like
EG01_15G015020	5	2	0.00025145	647	0.00010216	laccase-7-like
EG01_07G008440	11	2	0.00023708	3852	0.00010593	E3 ubiquitin-protein ligase PUB23-like
EG01_U01G003040	15	2	0.00023653	45440	0.00010556	leucine-rich repeat receptor-like serine
EG01_01G002640	5	1	0.00023549	0	0.00010213	inositol oxygenase 1-like
EG01_01G004970	5	1	0.00023549	0	0.00010213	ABC transporter C family member 10-like
EG01_U01G009620	26	5	0.00023119	27824	0.00009901	adenine phosphoribosyltransferase 1 isoform X2
EG01_U03G008660	11	3	0.00022794	1293	0.00010473	cysteine-rich repeat secretory protein 55-like
EG01_04G001960	9	1	0.00022446	0	0.00010327	pathogenesis-related protein 1-like
EG01_U01G033870	17	5	0.00022076	33043	0.00010416	pathogenesis-related protein 1-like
EG01_04G020200	25	1	0.00021748	0	0.00010381	leucine-rich repeat receptor-like tyrosine-protein kinase At2g41820
EG01_U01G008290	25	1	0.00021748	0	0.00010381	Type II proteinase inhibitor family protein
EG01_U02G007570	25	1	0.00021748	0	0.00010381	glucan endo-1%2C3-beta-glucosidase-like

EG01_U02G021740	25	1	0.00021748	0	0.00010381	pathogenesis-related protein 1-like
EG01_13G003470	5	2	0.00020176	1292	0.00010218	MLO-like protein 2%2C partial
EG01_U01G037860	5	2	0.00020084	647	0.00010216	calcium-binding protein KIC-like
EG01_U02G008230	11	1	0.00019899	0	0.00010469	premnaspirodiene oxygenase-like
EG01_04G004670	9	1	0.00018881	0	0.00010264	transcription factor IBH1
EG01_14G007050	5	1	0.00017248	0	0.00010197	Germin-like protein 8-2 OS%3DOryza sativa subsp. japonica GN%3DGER3 PE%3D2 SV%3D1
EG01_03G005620	2	3	0.00016845	1293	0.00007861	desiccation-related protein PCC13-62-like isoform X1
EG01_02G010370	25	2	0.00016807	647	0.00010302	Conserved hypothetical protein
EG01_11G008750	14	1	0.00016355	0	9.5529E-05	naringenin%2C2-oxoglutarate 3-dioxygenase-like
EG01_12G002850	25	1	0.00015739	0	0.000103	glucuronokinase 1 isoform X2
EG01_12G010840	25	1	0.00015739	0	0.000103	pectinesterase-like
EG01_08G011120	3	3	0.00014902	1937	9.6237E-05	protein NIM1-INTERACTING 3-like
EG01_U03G003400	2	1	0.00014706	0	7.8585E-05	trans-resveratrol di-O-methyltransferase-like
EG01_U02G003970	3	2	0.00013887	1292	9.6219E-05	LRR receptor-like serine threonine-protein kinase GSO1 VIT_11s0118g00160
EG01_11G001510	3	1	0.00012945	0	9.6182E-05	LRR receptor-like serine threonine-protein kinase GSO2 OsJ_05477
EG01_13G002110	3	3	0.0001263	1937	9.6089E-05	Doubtful hypothetical protein
EG01_U01G001720	21	3	0.00012365	83420	0.00011769	glycerophosphodiester phosphodiesterase GDPD6 isoform X2
EG01_02G010500	21	3	0.00011331	1293	0.00010114	WRKY transcription factor
EG01_U02G015940	21	1	0.0001045	0	0.00010468	metal transporter Nramp5-like
EG01_06G006990	21	1	9.7945E-05	0	0.00010106	Conserved hypothetical protein
EG01_13G005490	21	1	9.7945E-05	0	0.00010106	benzoate--CoA ligase%2C peroxisomal-like
EG01_13G004410	26	4	8.6229E-05	25641	9.3782E-05	photosystem I P700 apoprotein A1 (chloroplast)
EG01_U01G007990	20	3	8.6081E-05	8900	9.9671E-05	Doubtful hypothetical protein
EG01_U01G019930	20	3	8.5426E-05	1937	9.9453E-05	Conserved hypothetical protein
EG01_U01G039360	4	3	8.4983E-05	1937	9.9592E-05	Doubtful hypothetical protein
EG01_13G003690	24	2	8.3428E-05	647	7.8253E-05	luminal-binding protein 4-like
EG01_04G026080	4	2	8.0469E-05	13167	0.00009995	U-box domain-containing protein 27-like
EG01_U03G022670	11	3	7.9689E-05	3219	9.9236E-05	Conserved hypothetical protein
EG01_U01G018750	9	4	7.6711E-05	12034	9.7599E-05	protein kinase 2A%2C chloroplastic-like isoform X2
EG01_07G006170	25	3	6.8917E-05	1293	9.7428E-05	U-box domain-containing protein 26-like
EG01_09G009810	26	1	0.00006668	0	9.3049E-05	aspartic proteinase-like protein 1 isoform X2
EG01_11G013400	26	1	0.00006668	0	9.3049E-05	Conserved hypothetical protein
EG01_14G000150	26	1	0.00006668	0	9.3049E-05	cytochrome P450 86B1-like
EG01_12G003590	15	4	6.6055E-05	46711	0.00010038	phosphoinositide phospholipase C 6-like
EG01_13G009890	9	3	6.5872E-05	1293	9.6937E-05	hexose carrier protein HEX6-like
EG01_13G003230	11	1	6.5743E-05	0	9.8087E-05	PAR1 protein
EG01_U01G033860	11	1	6.5743E-05	0	9.8087E-05	pathogenesis-related protein 1-like
EG01_U01G034890	25	2	6.4243E-05	647	9.7409E-05	cysteine-rich receptor-like protein kinase 10
EG01_15G013620	25	2	6.3506E-05	647	9.7371E-05	transcription activator GLK2-like isoform X2
EG01_01G003710	9	2	6.2502E-05	1292	9.7276E-05	peroxidase P7-like
EG01_U01G034870	17	4	6.1587E-05	8287	9.7828E-05	cysteine-rich receptor-like protein kinase 10
EG01_U01G016090	9	2	6.1405E-05	647	9.6918E-05	reticuline oxidase-like
EG01_U03G010840	25	1	6.0163E-05	0	0.00009739	MDIS1-interacting receptor like kinase 2 OS%3DArabidopsis thaliana GN%3DMIK2 PE%3D1 SV%3D3
EG01_10G012080	25	1	5.9473E-05	0	9.7352E-05	acidic endochitinase-like
EG01_U02G024000	25	1	5.9473E-05	0	9.7352E-05	Sterile alpha motif domain-containing protein
EG01_U02G033080	17	3	5.6654E-05	9545	9.7867E-05	Hypothetical protein
EG01_05G004870	5	1	5.5367E-05	0	9.5822E-05	BTB POZ domain-containing protein At3g50780-like DCAR_002591
EG01_U02G001930	19	2	5.2863E-05	13772	9.8001E-05	Orf116d
EG01_U01G012410	17	1	4.8609E-05	0	0.00009758	trans-resveratrol di-O-methyltransferase-like
EG01_13G010710	5	2	4.7438E-05	647	9.5859E-05	Conserved hypothetical protein
EG01_U01G036800	5	1	4.4221E-05	0	9.5822E-05	protein YLS9
EG01_06G000590	2	1	0.00003709	0	7.4806E-05	gibberellin 3-beta-dioxygenase 1-like
EG01_13G000060	2	1	0.00003709	0	7.4806E-05	late embryogenesis abundant protein D-29-like
EG01_U03G023390	25	1	3.7006E-05	0	9.6581E-05	senescence-specific cysteine protease SAG39-like
EG01_U01G040610	9	7	0.00003604	7677	9.2005E-05	glutathione S-transferase U17-like
EG01_09G001250	3	2	3.5038E-05	647	9.0613E-05	ABC transporter B family member 11-like
EG01_01G015700	3	1	3.2813E-05	0	9.0596E-05	U-box domain-containing protein 21-like

EG01_U03G017200	23	3	3.2805E-05	86687	0.00011366	pathogenesis-related protein PRB1-3-like
EG01_02G006820	3	2	3.2651E-05	647	9.0596E-05	anthranilate O-methyltransferase 1-like
EG01_U02G029300	3	2	2.9696E-05	647	9.0481E-05	S-linalool synthase-like
EG01_U03G015090	21	2	2.9072E-05	647	0.00010939	Doubtful hypothetical protein
EG01_03G004070	26	6	2.8418E-05	22196	8.8984E-05	Putative methyltransferase DDB_G0268948
EG01_U02G000360	3	1	0.00002781	0	9.0465E-05	glutathione S-transferase U18-like
EG01_U03G022720	9	3	2.5615E-05	2579	0.00009187	ATP synthase CF1 alpha subunit (chloroplast)
EG01_05G003380	21	1	0.00002495	0	9.4931E-05	Conserved hypothetical protein
EG01_08G023210	21	1	0.00002495	0	9.4931E-05	Doubtful hypothetical protein
EG01_13G009550	4	4	2.3041E-05	12672	9.4233E-05	Doubtful hypothetical protein
EG01_10G003580	15	3	2.2316E-05	6404	9.4438E-05	flavonol synthase flavanone 3-hydroxylase-like MIMGU_mgv1a009032mg
EG01_U01G008680	17	4	0.00002221	5771	9.2157E-05	protein YLS9-like
EG01_08G000490	9	1	2.2125E-05	0	9.1802E-05	gibberellin 3-beta-dioxygenase 1
EG01_11G010780	11	4	2.1676E-05	1938	9.3301E-05	malonyl-coenzyme A:anthocyanin 3-O-glucoside-6"-O-malonyltransferase-like
EG01_01G014130	20	2	2.0928E-05	7007	9.3826E-05	transcription factor ILI5-like
EG01_U01G025350	15	2	2.0576E-05	13167	9.4634E-05	cytochrome P450 94B3-like
EG01_15G008480	15	2	2.0574E-05	27135	9.5066E-05	expansin-A4-like
EG01_02G011010	26	2	2.0374E-05	1935	8.8464E-05	cation transport regulator-like protein 2
EG01_13G011080	20	2	2.0239E-05	647	0.00009365	endoglucanase 1-like
EG01_10G011900	20	2	2.0085E-05	647	9.3458E-05	hevamine-A-like
EG01_U01G018840	4	2	1.9981E-05	647	0.00009358	thaumatin-like protein 1b
EG01_04G011510	25	1	1.9877E-05	0	9.1651E-05	Conserved hypothetical protein
EG01_08G007970	25	1	1.9877E-05	0	9.1651E-05	mannan endo-1%2C4-beta-mannosidase 1
EG01_10G014410	19	4	0.00001932	13262	9.2515E-05	palmitoyl-acyl carrier protein thioesterase%2C chloroplastic-like
EG01_05G007320	9	2	1.9249E-05	647	9.1533E-05	basic blue protein-like
EG01_08G013210	9	1	1.8999E-05	0	9.1216E-05	WAT1-related protein At5g64700-like isoform X1
EG01_U03G017980	9	1	1.8999E-05	0	9.1216E-05	Conserved hypothetical protein
EG01_U02G007860	26	1	1.8986E-05	0	8.8417E-05	Hexosyltransferase
EG01_U01G001570	17	2	1.8967E-05	647	9.2022E-05	Putative methylesterase 11%2C chloroplastic isoform X1
EG01_02G013570	17	3	1.8911E-05	5765	9.2191E-05	RING-H2 finger protein ATL13-like
EG01_01G014090	20	1	0.00001881	0	0.00009344	sucrose transport protein SUT3 isoform X1
EG01_04G013000	4	1	1.8712E-05	0	9.3563E-05	Doubtful hypothetical protein
EG01_U03G022490	25	1	1.8529E-05	0	9.1634E-05	Putative WRKY transcription factor 51 isoform X1
EG01_13G005900	24	1	0.00001837	0	7.4482E-05	uncharacterized transporter YBR287W-like
EG01_U01G006300	25	1	1.8316E-05	0	0.0000916	Putative protein
EG01_U03G000020	17	1	1.7763E-05	0	9.2005E-05	GST6 protein
EG01_U01G040190	9	1	0.00001771	0	9.1199E-05	mitoferrin
EG01_U01G040600	17	2	1.7629E-05	2576	9.2106E-05	Doubtful hypothetical protein
EG01_U03G019000	11	1	1.7546E-05	0	9.3249E-05	Protein SUPPRESSOR OF NPR1-1 CONSTITUTIVE 4 OS%3DArabidopsis thaliana GN%3DLRK10L-2.6 PE%3D1 SV%3D1
EG01_15G005160	5	1	1.3682E-05	0	9.0261E-05	Conserved hypothetical protein
EG01_U01G016340	23	5	1.3312E-05	19462	0.00010658	copper transporter 1-like
EG01_07G008180	16	4	1.2024E-05	73737	0.00010911	vacuolar iron transporter homolog 4-like
EG01_04G005630	3	1	1.0106E-05	0	8.5594E-05	WRKY transcription factor 79
EG01_05G000360	26	3	9.4949E-06	18569	8.4567E-05	Doubtful hypothetical protein
EG01_12G005280	3	1	9.4171E-06	0	0.00008558	Putative WRKY transcription factor 70
EG01_14G008440	20	6	8.9668E-06	6414	8.8613E-05	WRKY transcription factor 22-like
EG01_U02G032750	26	2	8.7521E-06	647	8.4154E-05	Doubtful hypothetical protein
EG01_U02G002960	9	2	8.5698E-06	3852	8.6926E-05	polyphenol oxidase%2C chloroplastic-like
EG01_07G003380	3	1	8.5649E-06	0	8.5477E-05	Conserved hypothetical protein
EG01_U01G031790	21	1	8.3849E-06	0	0.00010216	G-type lectin S-receptor-like serine threonine-protein kinase B120 isoform X2 RLK14
EG01_02G013120	4	4	8.2094E-06	1938	8.8865E-05	WRKY transcription factor 79
EG01_08G019260	4	4	8.2094E-06	1938	8.8865E-05	alpha-1%2C4 glucan phosphorylase L isozyme%2C chloroplastic amyloplastic SETIT_034019mg
EG01_08G022060	26	1	8.1963E-06	0	0.00008414	Glycosyl transferase family 2 family protein
EG01_13G003310	26	1	8.1963E-06	0	0.00008414	O-glucosyltransferase rumi homolog
EG01_U03G010980	26	1	8.1963E-06	0	0.00008414	crocetin glucosyltransferase%2C chloroplastic-like
EG01_01G015570	9	1	7.9355E-06	0	8.6836E-05	Conserved hypothetical protein

EG01_04G026840	9	1	7.9355E-06	0	8.6836E-05	subtilisin-like protease SBT3.3
EG01_09G004900	9	1	7.9355E-06	0	8.6836E-05	Linoleate 9S-lipoxygenase A OS%3DSolanum lycopersicum GN%3DLOX1.1 PE%3D2 SV%3D1
EG01_10G013870	9	1	7.9355E-06	0	8.6836E-05	WRKY transcription factor 42 OS%3DArabidopsis thaliana GN%3DWRKY42 PE%3D2 SV%3D1
EG01_15G012550	9	1	7.9355E-06	0	8.6836E-05	NAC domain-containing protein 100-like
EG01_01G001920	4	3	7.7549E-06	7035	8.8992E-05	Putative transcription factor bHLH041
EG01_12G003680	9	3	6.4606E-06	1293	8.6745E-05	protein SRG1-like
EG01_U02G032490	26	2	6.3038E-06	1292	8.3703E-05	photosystem II CP43 chlorophyll apoprotein (chloroplast)
EG01_03G008580	11	1	6.2518E-06	0	8.7989E-05	glutathione transferase GST 23-like
EG01_14G003210	11	1	6.2518E-06	0	8.7989E-05	Doubtful hypothetical protein
EG01_U02G009020	11	1	6.2518E-06	0	8.7989E-05	Putative protein phosphatase 2C 77 isoform X2
EG01_04G001620	20	1	5.8373E-06	0	0.0000883	Conserved hypothetical protein
EG01_09G007830	20	1	5.7929E-06	0	8.8129E-05	3-ketoacyl-CoA synthase 10-like
EG01_U02G018600	4	1	5.7629E-06	0	8.8238E-05	Isoform 2 of Cysteine-rich receptor-like protein kinase 10 OS%3DArabidopsis thaliana GN%3DCRK10
EG01_10G009770	15	3	5.6607E-06	3219	8.9079E-05	Conserved hypothetical protein
EG01_04G007230	15	3	5.6563E-06	1937	8.9047E-05	Doubtful hypothetical protein
EG01_14G000380	9	1	0.00000564	0	8.6715E-05	WD repeat-containing protein 44-like
EG01_02G009400	17	2	5.3092E-06	3215	8.7047E-05	phytosulfokines-like
EG01_16G006720	8	3	5.2846E-06	12624	8.9493E-05	Isoform 2 of Indole-3-acetic acid-amido synthetase GH3.2 OS%3DArabidopsis thaliana GN%3DGH3.2
EG01_01G017830	15	3	5.2784E-06	26736	9.0269E-05	cationic peroxidase 1-like
EG01_11G013990	17	2	5.2221E-06	647	8.6987E-05	Conserved hypothetical protein
EG01_09G003200	19	3	4.9695E-06	8889	8.7504E-05	E3 ubiquitin-protein ligase RNF126 isoform X1
EG01_01G009630	19	3	4.8987E-06	2579	8.7352E-05	non-specific lipid-transfer protein 2-like
EG01_10G011080	17	1	4.8904E-06	0	8.6972E-05	Conserved hypothetical protein
EG01_U02G005870	17	2	4.4687E-06	3852	8.7093E-05	Germin-like protein 12-2 OS%3DOryza sativa subsp. japonica GN%3DOs12g0154800 PE%3D2 SV%3D1
EG01_04G027630	17	3	4.4684E-06	1937	8.6972E-05	Pectin lyase-like superfamily protein isoform 2
EG01_04G009630	17	2	4.4464E-06	647	8.7017E-05	A-kinase anchor protein 9%2C Putative isoform 2
EG01_U02G034010	19	1	4.2539E-06	0	8.7291E-05	non-specific lipid-transfer protein 1-like
EG01_01G014370	9	1	4.2384E-06	0	8.6415E-05	cytochrome P450 71A1-like
EG01_02G013520	17	1	4.1763E-06	0	8.6851E-05	Doubtful hypothetical protein
EG01_U03G009820	23	4	3.6984E-06	7653	0.00009994	photosystem I P700 apoprotein A1 (chloroplast)
EG01_U02G021880	23	3	3.3576E-06	1293	9.9741E-05	sugar transport protein 13
EG01_U03G011050	10	3	3.152E-06	62793	0.00010441	cysteine-rich repeat secretory protein 55-like
EG01_U03G006680	23	2	3.1482E-06	3852	0.00009982	premnaspirodiene oxygenase-like
EG01_12G012580	23	2	3.1469E-06	4487	0.00009984	polygalacturonase At1g48100-like
EG01_12G012010	16	3	3.0864E-06	12651	0.00010233	GDSL esterase lipase At4g16230 isoform X3 JCGZ_19011
EG01_09G008740	9	3	2.8806E-06	3219	8.2366E-05	glutathione S-transferase U10-like
EG01_12G010850	16	1	2.6476E-06	0	0.00010192	endoglucanase 3 isoform X1
EG01_04G010710	26	3	2.4118E-06	17387	8.0541E-05	Doubtful hypothetical protein
EG01_12G002990	20	2	2.1301E-06	2576	8.3864E-05	Conserved hypothetical protein
EG01_U02G022890	20	2	2.1082E-06	647	8.3822E-05	Transcription factor WRKY33
EG01_09G009770	26	1	2.0906E-06	0	0.00008018	low affinity sulfate transporter 3-like
EG01_06G013330	4	3	2.0056E-06	3857	8.4232E-05	photosystem II 10 kDa polypeptide%2C chloroplastic-like
EG01_02G013920	20	1	1.9744E-06	0	8.3808E-05	ethylene-responsive transcription factor ERF003-like
EG01_04G004590	20	1	1.9744E-06	0	8.3808E-05	Doubtful hypothetical protein
EG01_11G010580	20	1	1.9744E-06	0	8.3808E-05	alpha-1%2C2-galactosyltransferase gmh3-like isoform X2
EG01_10G011020	26	1	1.9271E-06	0	7.9808E-05	pathogen-related protein isoform X1
EG01_U03G020070	17	4	1.9018E-06	2581	8.2461E-05	lysine histidine transporter 1-like
EG01_01G016740	9	1	1.8633E-06	0	8.2136E-05	calcium-transporting ATPase 8%2C plasma membrane-type-like isoform X1
EG01_15G000540	9	1	1.8633E-06	0	8.2136E-05	transcription factor bHLH92
EG01_16G004430	4	2	1.8415E-06	1935	8.4189E-05	mitochondrial phosphate carrier protein 3%2C mitochondrial-like
EG01_U01G009210	19	4	1.8169E-06	7661	8.2981E-05	dof zinc finger protein DOF5.6-like isoform X2
EG01_01G003370	4	1	1.8076E-06	0	8.4034E-05	heat stress transcription factor C-2b-like
EG01_02G002120	4	1	1.8076E-06	0	8.4034E-05	salutaridinol 7-O-acetyltransferase-like

EG01_11G004950	4	1	1.8076E-06	0	8.4034E-05	expansin-B18-like
EG01_14G001140	4	1	1.8076E-06	0	8.4034E-05	mechanosensitive ion channel protein 6-like
EG01_U02G021300	4	1	1.8076E-06	0	8.4034E-05	Doubtful hypothetical protein
EG01_U02G023430	4	1	1.8076E-06	0	8.4034E-05	Conserved hypothetical protein
EG01_U02G027210	8	3	1.7892E-06	9531	0.00008481	L-type lectin-domain containing receptor kinase IV.2-like
EG01_01G002870	7	3	1.7651E-06	23789	8.5859E-05	Conserved hypothetical protein
EG01_12G000740	15	2	1.7607E-06	1935	8.4267E-05	laccase-25-like%2C partial
EG01_U02G017880	15	2	1.742E-06	647	8.4211E-05	anthocyanidin 5%2C3-O-glucosyltransferase-like
EG01_U01G027160	8	2	1.6356E-06	1935	8.4638E-05	estradiol 17-beta-dehydrogenase 8-like
EG01_12G008860	15	2	1.6337E-06	1935	8.5332E-05	uclacyanin-2-like
EG01_15G015540	15	1	1.6327E-06	0	8.4225E-05	protein NRT1, PTR FAMILY 8.1-like
EG01_13G002830	15	1	1.6314E-06	0	8.4196E-05	Doubtful hypothetical protein
EG01_06G007110	19	2	1.5157E-06	1292	8.2706E-05	transcription factor mef2A-like
EG01_10G014420	17	1	1.5061E-06	0	8.2352E-05	pleiotropic drug resistance protein 3-like
EG01_04G013020	26	2	1.4821E-06	647	7.9416E-05	limonoid UDP-glucosyltransferase-like
EG01_05G001600	19	1	1.4333E-06	0	8.2816E-05	Putative receptor-like protein kinase At1g33260
EG01_02G001990	19	1	1.4129E-06	0	8.2679E-05	protein CHLOROPLAST IMPORT APPARATUS 2-like isoform X1
EG01_09G002610	17	2	1.3836E-06	3215	8.2515E-05	LRR-RLK
EG01_U02G015150	17	2	1.3762E-06	647	8.2352E-05	cytokinin dehydrogenase 3-like
EG01_U03G012330	23	4	1.3511E-06	5774	9.4029E-05	receptor-like protein kinase HSL1
EG01_03G003980	17	1	1.2888E-06	0	8.2338E-05	NAC transcription factor 29-like
EG01_06G001710	17	1	1.2824E-06	0	8.2379E-05	L-ascorbate oxidase
EG01_U03G002200	10	4	1.1547E-06	60107	0.00009995	WAT1-related protein At5g07050-like
EG01_01G006360	10	4	1.136E-06	3222	9.7895E-05	LOB domain-containing protein 11-like
EG01_12G011290	23	1	1.0667E-06	0	0.00009387	polyphenol oxidase%2C chloroplastic-like
EG01_15G003450	23	1	1.0667E-06	0	0.00009387	pectinesterase-like
EG01_12G009330	16	3	1.0316E-06	7664	0.0000962	thaumatin-like protein
EG01_07G006320	23	2	9.8574E-07	3215	9.3853E-05	amino-acid permease BAT1 homolog
EG01_02G003810	23	2	9.8005E-07	3852	9.3888E-05	1-aminocyclopropane-1-carboxylate oxidase-like
EG01_15G000410	23	1	9.6839E-07	0	9.3694E-05	glu S.griseus protease inhibitor-like
EG01_U02G022710	23	1	9.6839E-07	0	9.3694E-05	tetrahydrocannabinolic acid synthase-like
EG01_U03G019870	16	2	9.6132E-07	3852	9.6089E-05	Conserved hypothetical protein
EG01_04G016430	9	4	7.8355E-07	1938	7.8235E-05	Conserved hypothetical protein
EG01_07G001300	4	5	7.7544E-07	2582	0.00007993	Conserved hypothetical protein
EG01_05G003630	26	2	7.6295E-07	16172	7.6858E-05	chitinase 1-like
EG01_U01G036870	20	3	7.0722E-07	1937	7.9586E-05	cytochrome P450 71A1-like
EG01_U02G000790	26	1	6.9561E-07	0	7.6552E-05	ABC transporter C family member 10-like
EG01_U01G017470	9	1	6.3427E-07	0	7.8198E-05	tetrahydrocannabinolic acid synthase-like
EG01_U02G013050	4	3	6.0839E-07	1293	7.9866E-05	22.7 kDa class IV heat shock protein-like
EG01_04G015900	20	1	6.0805E-07	0	0.00007951	Doubtful hypothetical protein
EG01_U02G025610	4	1	5.7846E-07	0	7.9879E-05	Protein phosphatase 2C 3 OS%3DArabidopsis thaliana GN%3DAIP1 PE%3D1 SV%3D1
EG01_14G004500	19	4	5.0264E-07	5140	7.8852E-05	flavanone 3-dioxygenase
EG01_02G012770	8	4	4.9638E-07	7026	8.0541E-05	spermidine synthase 1-like
EG01_06G009740	7	3	4.5287E-07	22739	8.1833E-05	EG45-like domain containing protein
EG01_01G007750	17	2	4.4714E-07	647	7.8296E-05	cytosolic sulfotransferase 12-like
EG01_13G002570	15	3	4.441E-07	1293	7.9936E-05	zinc finger protein ZAT5-like
EG01_08G010490	26	1	4.2747E-07	0	7.5534E-05	neurofilament medium polypeptide-like
EG01_U01G032010	19	2	4.2717E-07	647	7.8765E-05	glucomannan 4-beta-mannosyltransferase 9-like
EG01_14G002330	8	2	4.2262E-07	1292	8.0425E-05	ethylene-responsive transcription factor 1B-like
EG01_08G004510	17	1	4.1874E-07	0	7.8284E-05	Conserved hypothetical protein
EG01_14G004310	17	1	4.1874E-07	0	7.8284E-05	heavy metal-associated isoprenylated plant protein 26-like
EG01_06G002250	19	1	4.0005E-07	0	7.8753E-05	receptor-like protein kinase HSL1
EG01_03G003120	7	1	3.8865E-07	0	0.00008134	sex determination protein tasselseed-2-like
EG01_U02G033270	8	2	3.8633E-07	1292	0.00008027	Conserved hypothetical protein
EG01_10G007790	15	2	3.8588E-07	1292	8.0893E-05	F-box protein At1g61340-like
EG01_U03G022730	15	1	3.8357E-07	0	7.9859E-05	ATP synthase CF0 subunit I (chloroplast)
EG01_06G004370	23	4	3.6947E-07	2581	0.0000887	BURP domain-containing protein 3-like
EG01_12G012760	19	2	3.5636E-07	647	7.8518E-05	S-adenosylmethionine synthase
EG01_15G013310	17	2	3.2869E-07	2576	7.8382E-05	photosystem II CP43 chlorophyll apoprotein (chloroplast)

EG01_10G011930	23	2	3.1913E-07	1292	8.8668E-05	S-type anion channel SLAH2-like
EG01_08G006230	17	1	3.0301E-07	0	7.8186E-05	sugar transport protein 14-like
EG01_04G019050	23	1	2.9749E-07	0	8.8637E-05	2-dehydro-3-deoxyphosphooctonate aldolase
EG01_04G005950	10	3	2.9631E-07	3219	9.3967E-05	acidic mammalian chitinase-like
EG01_U03G005220	10	3	2.8652E-07	1293	9.2098E-05	chalcone synthase-like
EG01_U03G011180	10	2	2.8253E-07	54800	9.5675E-05	glucan endo-1%2C3-beta-glucosidase-like%2C partial
EG01_07G004430	10	2	2.7274E-07	1292	9.3914E-05	endochitinase A-like isoform X2
EG01_U03G010590	23	4	2.6956E-07	2581	8.8543E-05	isoflavone reductase-like protein
EG01_04G012000	23	3	2.5108E-07	3221	0.00008859	1-aminocyclopropane-1-carboxylate oxidase 3
EG01_U02G003640	10	1	2.5013E-07	0	9.2064E-05	glycine-rich cell wall structural protein 2-like
EG01_U03G006000	10	1	2.5013E-07	0	9.2064E-05	chalcone synthase-like
EG01_03G006390	16	3	2.4669E-07	3219	9.0547E-05	glucan endo-1%2C3-beta-glucosidase-like
EG01_07G006630	16	2	2.466E-07	4487	9.0678E-05	short-chain dehydrogenase TIC 32%2C chloroplastic-like
EG01_U02G021820	16	2	2.4375E-07	1935	9.0613E-05	3-ketoacyl-CoA synthase 11-like
EG01_04G013710	22	5	2.3351E-07	15700	7.3486E-05	beta-glucosidase 12-like isoform X3
EG01_07G000920	9	1	2.2599E-07	0	7.4466E-05	protein TIFY 9
EG01_16G003060	9	1	2.2599E-07	0	7.4466E-05	L-ascorbate oxidase-like
EG01_U01G003080	9	1	2.2599E-07	0	7.4466E-05	leucine-rich repeat receptor-like serine
EG01_12G004110	8	4	1.7884E-07	5137	7.6646E-05	zinc transporter 8-like isoform X2
EG01_05G005750	4	1	1.7074E-07	0	7.5999E-05	histone H2B.11-like
EG01_06G012140	4	1	1.7074E-07	0	7.5999E-05	Copper transport protein ATX1 OS%3DArabidopsis thaliana GN%3DATX1 PE%3D1 SV%3D2
EG01_12G000910	4	1	1.7074E-07	0	7.5999E-05	Lipid binding protein
EG01_U02G021530	4	1	1.7074E-07	0	7.5999E-05	Os01g0206650 protein
EG01_08G016280	20	2	1.6628E-07	647	0.0000757	1-deoxy-D-xylulose-5-phosphate synthase 1%2C chloroplastic OS%3DOryza sativa subsp. japonica GN%3DCLA1 PE%3D2 SV%3D2
EG01_04G019380	19	2	1.5634E-07	1935	7.5058E-05	12-oxophytodienoate reductase 2 OS%3DArabidopsis thaliana GN%3DOPR2 PE%3D1 SV%3D2
EG01_U02G013500	20	1	1.5572E-07	0	7.5689E-05	UDP-glycosyltransferase 89B1-like
EG01_U01G036150	19	2	1.548E-07	647	7.5036E-05	protein FEZ-like
EG01_U03G026880	19	2	1.548E-07	647	7.5036E-05	malate synthase%2C glyoxysomal
EG01_U01G032140	7	3	1.5036E-07	1937	7.7754E-05	Putative WRKY transcription factor 70
EG01_13G005380	8	1	1.4317E-07	0	7.6552E-05	PH domain-containing protein DDB_G0287875-like
EG01_U03G018230	8	1	1.4317E-07	0	7.6552E-05	Doubtful hypothetical protein
EG01_10G007060	7	2	1.413E-07	19712	7.8107E-05	Indole-3-acetic acid-amido synthetase GH3.6 OS%3DArabidopsis thaliana GN%3DGH3.6 PE%3D1 SV%3D1
EG01_08G018750	4	1	1.3396E-07	0	7.5942E-05	Perakine reductase OS%3DRauvolfia serpentina GN%3DPR PE%3D1 SV%3D1
EG01_12G003940	4	1	1.3396E-07	0	7.5942E-05	ammonium transporter 2 member 1-like
EG01_03G002330	8	2	1.3016E-07	647	7.6458E-05	flavonol synthase flavanone 3-hydroxylase-like isoform X2 PRUPE_ppa007719mg
EG01_14G003230	17	1	1.2896E-07	0	7.4521E-05	Putative glycine-rich cell wall structural protein 1
EG01_U01G008000	10	6	1.2846E-07	55188	9.1735E-05	pathogenesis-related protein 1-like
EG01_01G007390	15	1	1.2808E-07	0	7.6005E-05	pathogen-related protein-like
EG01_U01G014830	15	1	1.2808E-07	0	7.6005E-05	sugar transporter ERD6-like 16
EG01_15G010470	19	1	1.232E-07	0	7.4946E-05	xyloglucan endotransglucosylase hydrolase protein 9-like EUGRSUZ_K00883
EG01_U01G024690	8	2	1.1898E-07	647	7.6318E-05	Doubtful hypothetical protein
EG01_05G002130	15	2	1.1884E-07	647	7.6882E-05	naringenin%2C2-oxoglutarate 3-dioxygenase-like
EG01_U03G004140	23	2	1.1379E-07	647	0.0000839	zinc transporter 8-like isoform X2
EG01_01G018310	17	3	1.0913E-07	1937	7.4632E-05	Doubtful hypothetical protein
EG01_04G017830	23	1	1.0656E-07	0	8.3886E-05	Doubtful hypothetical protein
EG01_10G009620	23	1	1.0656E-07	0	8.3886E-05	dirigent protein 19-like
EG01_11G008230	10	4	1.0557E-07	1938	8.8629E-05	Putative Expressed protein
EG01_U02G027860	19	1	1.0278E-07	0	7.4722E-05	chalcone synthase 3-like
EG01_U02G033760	23	2	9.8284E-08	647	8.3872E-05	Putative B12D-like
EG01_05G002270	16	4	8.837E-08	3861	8.5741E-05	reticuline oxidase-like
EG01_08G013200	16	4	8.7895E-08	1938	0.00008558	WAT1-related protein At5g64700-like isoform X1
EG01_01G016840	22	3	8.6034E-08	11420	7.0328E-05	Conserved hypothetical protein
EG01_03G004480	10	1	8.5461E-08	0	8.8582E-05	Doubtful hypothetical protein
EG01_04G005340	10	2	8.3998E-08	647	0.00008855	phospholipase D alpha 1-like

EG01_06G006390	23	2	8.3019E-08	647	8.3759E-05	microsomal glutathione S-transferase 3-like
EG01_U03G023680	23	3	8.2953E-08	1293	8.3815E-05	beta-glucosidase 18-like%2C partial
EG01_04G014600	10	1	8.2638E-08	0	8.6919E-05	DUF506 family protein
EG01_U03G016110	10	1	8.2638E-08	0	8.6919E-05	pathogenesis-related protein PR-1 type-like
EG01_08G001530	23	1	7.7747E-08	0	8.3745E-05	Putative WRKY transcription factor 72
EG01_08G004270	23	1	7.7747E-08	0	8.3745E-05	S-type anion channel SLAH2-like
EG01_09G012090	23	2	7.7328E-08	647	8.3801E-05	alpha-amylase isozyme 3D-like
EG01_15G010640	16	2	7.5415E-08	1292	8.5624E-05	aromatic-L-amino-acid decarboxylase-like
EG01_12G015650	22	2	7.2246E-08	1292	7.0171E-05	ankyrin repeat-containing protein At5g02620-like
EG01_06G004200	22	2	7.1914E-08	647	7.0161E-05	BURP domain-containing protein 6-like
EG01_06G013880	16	1	7.1149E-08	0	8.5536E-05	Conserved hypothetical protein
EG01_13G008210	22	1	6.7347E-08	0	7.0151E-05	Doubtful hypothetical protein
EG01_09G012000	20	1	4.7958E-08	0	7.2166E-05	galactoside 2-alpha-L-fucosyltransferase-like
EG01_10G012070	13	6	4.5458E-08	51728	8.7951E-05	hevamine-A-like
EG01_04G017740	22	10	4.4214E-08	5787	6.7349E-05	Conserved hypothetical protein
EG01_U01G011140	8	2	4.2257E-08	2576	7.3067E-05	Perakine reductase OS%3DRauvolfia serpentina GN%3DPR PE%3D1 SV%3D1
EG01_11G013210	8	2	4.2048E-08	647	7.3035E-05	chitinase 1-like
EG01_U01G032130	19	3	3.9433E-08	1293	7.1602E-05	Putative WRKY transcription factor 70
EG01_U01G005670	8	1	3.9378E-08	0	7.3025E-05	Conserved hypothetical protein
EG01_U01G013930	7	3	3.7029E-08	19257	7.4694E-05	premnaspirodiene oxygenase-like
EG01_U02G032920	7	2	3.5352E-08	647	7.4041E-05	Glyceraldehyde-3-phosphate dehydrogenase 2%2C cytosolic (Fragment) OS%3DHordeum vulgare GN%3DGAPC PE%3D2 SV%3D1
EG01_15G007800	19	1	3.4085E-08	0	7.1561E-05	protein PHLOEM PROTEIN 2-LIKE A1
EG01_U01G008630	19	1	3.4085E-08	0	7.1561E-05	Basic secretory protease (Fragments) OS%3DBoswellia serrata PE%3D1 SV%3D1
EG01_10G012110	7	1	3.3107E-08	0	0.00007403	acidic endochitinase-like
EG01_01G003720	10	2	3.0505E-08	1935	0.00008664	peroxidase P7-like
EG01_14G003240	10	2	3.0344E-08	1292	8.6625E-05	Doubtful hypothetical protein
EG01_U03G003830	8	1	2.8659E-08	0	7.2854E-05	expansin-like B1
EG01_09G005370	10	1	2.8286E-08	0	8.6595E-05	mitochondrial chaperone BCS1-like
EG01_U03G008530	10	1	2.8286E-08	0	8.6595E-05	glucan endo-1%2C3-beta-glucosidase-like%2C partial
EG01_01G003190	8	1	2.6198E-08	0	7.2727E-05	DNA binding protein%2C Putative
EG01_12G016260	15	1	2.6167E-08	0	7.3239E-05	Conserved hypothetical protein
EG01_02G000740	17	2	2.5658E-08	647	7.1205E-05	Aldose reductase
EG01_U02G012100	23	1	2.5054E-08	0	0.00007958	calcium uniporter protein 4%2C mitochondrial-like
EG01_U03G005580	17	1	2.4029E-08	0	7.1195E-05	Cytochrome c oxidase subunit 1
EG01_06G004060	10	1	2.3246E-08	0	8.3822E-05	BAG family molecular chaperone regulator 6
EG01_08G017200	10	1	2.3246E-08	0	8.3822E-05	Glycosyl transferase family 2 protein
EG01_09G010880	10	1	2.3246E-08	0	8.3822E-05	Putative Os04g0450600 protein (Fragment)
EG01_U02G012140	23	1	2.1641E-08	0	7.9554E-05	Ribosomal protein S7%2C mitochondrial OS%3DTriticum aestivum GN%3DRPS7 PE%3D3 SV%3D1
EG01_U01G029000	16	2	2.088E-08	1935	8.1274E-05	glutathione S-transferase 3-like%2C partial
EG01_04G001600	22	2	2.0577E-08	4487	6.7331E-05	serine carboxypeptidase-like 33
EG01_15G005310	27	6	1.9748E-08	8316	8.3396E-05	cinnamoyl-CoA reductase 1-like
EG01_14G005960	16	1	1.9458E-08	0	8.1235E-05	pectinesterase-like
EG01_15G001000	16	1	1.9458E-08	0	8.1235E-05	protein jagged-1b-like
EG01_04G012740	16	1	1.9353E-08	0	0.00008109	oryzain alpha chain-like
EG01_08G022750	16	1	1.9353E-08	0	0.00008109	transcription factor JUNGBRUNNEN 1-like
EG01_U01G019680	16	1	1.9353E-08	0	0.00008109	thaumatin-like protein 1b
EG01_10G001830	10	1	1.8495E-08	0	8.3752E-05	chitinase 2-like
EG01_U03G014170	23	1	1.828E-08	0	7.9453E-05	cinnamoyl-CoA reductase 1-like
EG01_04G020860	23	1	1.8265E-08	0	7.9504E-05	serine decarboxylase 1
EG01_U01G016100	23	1	1.8265E-08	0	7.9504E-05	thaumatin-like protein
EG01_U02G031540	16	2	1.7731E-08	647	8.1142E-05	pathogenesis-related protein 1-like
EG01_U03G025490	23	1	1.7027E-08	0	7.9491E-05	DNA-directed RNA polymerase III subunit rpc6
EG01_16G007230	22	2	1.6986E-08	647	6.7132E-05	Conserved hypothetical protein
EG01_02G007010	13	4	1.6751E-08	7673	8.3382E-05	Exo-poly-alpha-D-galacturonosidase
EG01_11G014270	22	1	1.5834E-08	0	6.7114E-05	subtilisin-like protease SDD1
EG01_U02G002140	13	3	1.5268E-08	34919	8.4041E-05	ethylene-responsive transcription factor ERF113-like
EG01_08G022300	7	4	1.3613E-08	15622	7.1505E-05	receptor-like protein kinase HAIKU2
EG01_U02G027520	7	4	1.3264E-08	2581	7.1291E-05	Doubtful hypothetical protein

EG01_04G009620	13	1	1.3111E-08	0	8.3215E-05	Galactose mutarotase-like superfamily protein isoform 1
EG01_04G028250	13	1	1.3111E-08	0	8.3215E-05	Conserved hypothetical protein
EG01_U01G012050	8	2	1.3079E-08	1935	6.9798E-05	Putative protein
EG01_02G011360	22	1	1.2752E-08	0	6.4537E-05	11 kDa late embryogenesis abundant protein-like
EG01_04G004740	22	1	1.2752E-08	0	6.4537E-05	Doubtful hypothetical protein
EG01_05G004030	22	1	1.2752E-08	0	6.4537E-05	monosaccharide-sensing protein 2-like
EG01_08G012370	22	1	1.2752E-08	0	6.4537E-05	transcription factor MYB3-like
EG01_10G002520	22	1	1.2752E-08	0	6.4537E-05	ninja-family protein AFP3
EG01_11G010560	22	1	1.2752E-08	0	6.4537E-05	Protein phosphatase 2C 3 OS%3DArabidopsis thaliana GN%3DAIP1 PE%3D1 SV%3D1
EG01_U02G004120	22	1	1.2752E-08	0	6.4537E-05	serine threonine-protein kinase SAPK3-like RCOM_0855720
EG01_U02G008580	22	1	1.2752E-08	0	6.4537E-05	alpha-terpineol synthase%2C chloroplastic-like
EG01_U03G002100	22	1	1.2752E-08	0	6.4537E-05	Alpha-terpineol synthase%2C chloroplastic OS%3DMagnolia grandiflora PE%3D1 SV%3D1
EG01_U02G002810	8	1	1.2127E-08	0	0.00006974	Leucine Rich Repeat family protein%2C expressed
EG01_07G008940	19	1	1.1373E-08	0	6.8432E-05	NAC domain-containing protein 90-like
EG01_10G011570	19	1	1.1373E-08	0	6.8432E-05	Harpin-induced protein
EG01_U02G006940	7	1	1.0196E-08	0	7.0656E-05	chalcone synthase 3-like
EG01_16G003850	10	3	1.0078E-08	1293	8.2068E-05	Putative LRR receptor-like serine
EG01_U03G005530	10	2	9.3451E-09	647	8.2041E-05	pathogenesis-related protein 1-like
EG01_U01G038170	22	4	7.4196E-09	3861	0.00006457	Hypothetical protein
EG01_08G019820	17	1	7.4003E-09	0	6.8069E-05	cytochrome P450 81E8-like
EG01_10G010830	16	2	6.4603E-09	1292	7.7238E-05	inositol oxygenase 1-like
EG01_12G003880	16	1	5.114E-09	0	7.7095E-05	pyruvate decarboxylase 2-like
EG01_05G003640	27	3	5.0054E-09	1937	7.9164E-05	protein FEZ-like
EG01_01G005850	13	5	4.973E-09	3224	7.9177E-05	Conserved hypothetical protein
EG01_U03G004790	22	1	4.8992E-09	0	6.4338E-05	senescence-specific cysteine protease SAG39-like
EG01_11G007710	27	2	4.6661E-09	2576	7.9177E-05	geraniol 8-hydroxylase-like
EG01_06G007450	27	2	4.643E-09	647	7.9139E-05	Rust resistance kinase Lr10 OS%3DTriticum aestivum GN%3DLRK10 PE%3D2 SV%3D1
EG01_U02G013540	27	1	4.3481E-09	0	7.9126E-05	cysteine-rich receptor-like protein kinase 2
EG01_U02G030360	27	1	4.3481E-09	0	7.9126E-05	Germin-like protein 8-9 OS%3DOryza sativa subsp. japonica GN%3DOs08g0189850 PE%3D2 SV%3D1
EG01_U01G022690	12	5	4.1762E-09	13888	6.8549E-05	UDP-glucuronate:xylan alpha-glucuronosyltransferase 2-like isoform X2
EG01_U02G016230	13	2	3.9595E-09	2576	7.9164E-05	protein P21-like
EG01_16G001480	13	3	3.8716E-09	32669	8.0412E-05	peroxidase 15-like
EG01_U01G036640	13	1	3.6883E-09	0	7.9114E-05	L-gulonolactone oxidase-like
EG01_04G004360	13	2	3.6063E-09	1292	7.9732E-05	glutamate receptor 2.7-like
EG01_U03G024020	7	2	3.1186E-09	647	6.8157E-05	pathogenesis-related protein 1-like
EG01_04G008190	8	2	3.0892E-09	1292	0.0000668	3-oxoacyl-[acyl-carrier-protein] reductase%2C chloroplastic-like
EG01_15G002310	7	1	2.9975E-09	0	6.8343E-05	(R%2CS)-reticuline 7-O-methyltransferase-like
EG01_U01G027170	7	1	2.9975E-09	0	6.8343E-05	WRKY DNA binding protein
EG01_U02G013370	7	1	2.9206E-09	0	6.8148E-05	premnaspirodiene oxygenase-like
EG01_U03G007670	7	1	2.9206E-09	0	6.8148E-05	Doubtful hypothetical protein
EG01_U01G015280	10	1	2.2191E-09	0	0.00007793	cytochrome P450 71A1-like
EG01_U01G024320	10	1	2.2191E-09	0	0.00007793	two-component response regulator-like APRR9 isoform X3
EG01_08G001150	10	1	2.0577E-09	0	7.7906E-05	Doubtful hypothetical protein
EG01_09G009780	22	3	1.8807E-09	1937	6.2004E-05	bifunctional aspartate aminotransferase and glutamate aspartate-prephenate aminotransferase-like pco075539
EG01_06G007830	22	1	1.6337E-09	0	6.1981E-05	cysteine-rich repeat secretory protein 38-like
EG01_U02G022170	22	1	1.6337E-09	0	6.1981E-05	pathogenesis-related protein PRB1-3-like
EG01_06G009970	12	4	1.5508E-09	8927	6.5759E-05	EG45-like domain containing protein
EG01_U01G002760	27	2	1.5416E-09	647	7.5318E-05	phospholipase A1-Igamma1%2C chloroplastic-like
EG01_05G002140	13	2	1.5316E-09	647	0.00007533	flavonol synthase flavanone 3-hydroxylase-like CISIN_1g019027mg
EG01_01G007650	16	2	1.5189E-09	647	7.3573E-05	Doubtful hypothetical protein
EG01_09G005620	27	2	1.4442E-09	1935	7.5352E-05	carbonic anhydrase%2C chloroplastic-like
EG01_05G004210	27	1	1.4437E-09	0	7.5307E-05	benzoate--CoA ligase%2C peroxisomal-like

EG01_08G003770	13	1	1.4343E-09	0	7.5318E-05	zingipain-2-like
EG01_08G021460	13	1	1.4343E-09	0	7.5318E-05	glutathione S-transferase 3-like
EG01_U02G001700	13	1	1.4343E-09	0	7.5318E-05	WAT1-related protein At5g07050-like
EG01_09G002080	12	3	1.3935E-09	2580	6.5673E-05	ureide permease 1-like
EG01_U03G004270	27	1	1.3391E-09	0	7.5284E-05	potassium transporter 5-like
EG01_11G008820	13	2	1.2316E-09	1935	7.5341E-05	peroxidase 5-like
EG01_06G001260	12	1	1.2045E-09	0	6.5638E-05	WRKY transcription factor 42
EG01_12G009680	12	1	1.2045E-09	0	6.5638E-05	glucan endo-1%2C3-beta-glucosidase-like
EG01_11G004380	13	2	1.1989E-09	31535	7.7059E-05	NAC domain-containing protein 72-like isoform X1
EG01_01G011100	13	1	1.1166E-09	0	7.6435E-05	beta-xylosidase alpha-L-arabinofuranosidase 2-like VIT_07s0005g01300
EG01_09G009100	13	2	1.1106E-09	647	7.5832E-05	transcription factor ILI5-like
EG01_10G001810	8	2	9.5141E-10	647	6.4041E-05	chitinase 2-like
EG01_U03G017210	7	1	8.9946E-10	0	6.5278E-05	chitinase 2-like
EG01_09G009620	22	2	5.7919E-10	647	0.00005962	beta-xylosidase alpha-L-arabinofuranosidase 2-like OsI_08964
EG01_U01G007010	22	1	5.4241E-10	0	5.9613E-05	endoglucanase 17-like
EG01_U03G008540	12	5	4.6987E-10	5141	6.3139E-05	Inositol oxygenase 1 OS%3DArabidopsis thaliana GN%3DMIOX1 PE%3D2 SV%3D1
EG01_03G007780	16	1	4.3809E-10	0	0.00007023	endo-1%2C3%3B1%2C4-beta-D-glucanase-like
EG01_15G005210	12	2	3.6631E-10	1292	6.3091E-05	Lectin-like protein kinase
EG01_15G011520	12	2	3.6463E-10	647	6.3084E-05	cysteine proteinase inhibitor 12-like
EG01_11G011230	27	2	3.4112E-10	1292	0.00007187	caffeic acid 3-O-methyltransferase-like
EG01_07G004140	27	1	3.3943E-10	0	7.1818E-05	extensin
EG01_10G012390	13	1	3.3723E-10	0	7.1829E-05	Putative WRKY transcription factor 53
EG01_15G002180	12	2	3.2763E-10	647	6.3004E-05	non-functional NADPH-dependent codeinone reductase 2-like
EG01_16G003250	12	2	3.2763E-10	647	6.3004E-05	annexin D1-like
EG01_12G013400	13	3	3.1063E-10	1293	0.00007186	acid phosphatase 1
EG01_11G010030	13	2	2.8525E-10	30992	7.3964E-05	crocetin glucosyltransferase 3-like
EG01_04G020810	13	1	2.4455E-10	0	7.2286E-05	U-box domain-containing protein 27
EG01_08G011430	8	1	2.0949E-10	0	6.1493E-05	S-linalool synthase-like
EG01_11G014290	12	4	1.6742E-10	1938	6.0683E-05	subtilisin-like protease SDD1
EG01_12G015560	12	2	1.4471E-10	647	6.0669E-05	Putative LRR receptor-like serine
EG01_01G004400	12	1	1.3552E-10	0	6.0661E-05	GDSL esterase, lipase At5g45910-like
EG01_12G009320	12	1	1.3552E-10	0	6.0661E-05	thaumatin-like protein 1
EG01_10G014350	22	1	1.2753E-10	0	5.7405E-05	Conserved hypothetical protein
EG01_U01G025000	12	2	1.1281E-10	647	6.0624E-05	L-type lectin-domain containing receptor kinase IV.1-like
EG01_U03G005040	12	1	1.0516E-10	0	0.00006061	Putative Hop-interacting protein THI031
EG01_10G007510	27	2	1.0506E-10	647	6.8686E-05	AP2 ERF and B3 domain-containing transcription factor RAV1-like TCM_011862
EG01_16G003200	13	3	9.6593E-11	30635	7.1098E-05	Leucine Rich Repeat family protein
EG01_10G014230	12	1	9.4494E-11	0	6.0536E-05	Copper transport protein ATX1 OS%3DArabidopsis thaliana GN%3DATX1 PE%3D1 SV%3D2
EG01_U01G022850	12	1	9.4494E-11	0	6.0536E-05	Chavicol synthase
EG01_03G006380	13	1	8.9591E-11	0	6.8667E-05	glucan endo-1%2C3-beta-glucosidase-like
EG01_U01G003550	13	1	8.9591E-11	0	6.8667E-05	pathogenesis-related protein 5
EG01_06G003160	12	1	3.6862E-11	0	5.8391E-05	gibberellin 2-beta-dioxygenase 1-like
EG01_13G003070	12	1	3.6862E-11	0	5.8391E-05	chlorophyllase-1-like
EG01_U01G008500	12	1	3.6862E-11	0	5.8391E-05	glucuronoxylan 4-O-methyltransferase 1-like
EG01_15G007660	12	1	3.1863E-11	0	5.8377E-05	Conserved hypothetical protein
EG01_08G003490	18	3	2.5174E-11	27897	6.8399E-05	Conserved hypothetical protein
EG01_14G007070	12	1	2.484E-11	0	5.8336E-05	Rust resistance kinase Lr10 OS%3DTriticum aestivum GN%3DLRK10 PE%3D2 SV%3D1
EG01_14G000870	13	3	2.4483E-11	1937	6.7999E-05	lichenase-2-like
EG01_04G013980	27	1	2.3132E-11	0	6.5764E-05	DNA-damage-repair
EG01_12G013990	18	4	9.3333E-12	24467	6.5846E-05	protein LURP-one-related 10-like
EG01_11G003650	18	3	8.406E-12	2579	6.5535E-05	mitochondrial chaperone BCS1-like
EG01_15G000710	13	2	7.5403E-12	647	6.5142E-05	chitinase 2-like
EG01_U03G020240	13	1	7.0614E-12	0	6.5134E-05	cytochrome P450 71A1-like
EG01_U03G007070	18	5	2.9265E-12	21858	6.3436E-05	protein RSI-1-like
EG01_U02G032010	18	2	2.2045E-12	1292	6.3171E-05	heavy metal-associated isoprenylated plant protein 26-like

EG01_U02G028870	18	3	2.1202E-12	1293	6.2885E-05	sphinganine C(4)-monooxygenase 2-like
EG01_04G001910	18	1	2.0551E-12	0	6.3155E-05	pre-mRNA-splicing factor cwf23-like
EG01_14G000880	18	1	1.8509E-12	0	6.2869E-05	glucan endo-1%2C3-beta-glucosidase-like
EG01_U03G003700	13	1	1.6603E-12	0	6.2508E-05	scarecrow-like protein 3
EG01_04G008960	18	5	1.1385E-12	3864	6.0979E-05	Conserved hypothetical protein
EG01_U02G033240	18	3	9.8954E-13	7037	6.1017E-05	acidic endochitinase-like
EG01_11G001530	1	3	9.8597E-13	8889	6.1039E-05	LRR receptor-like serine, threonine-protein kinase GSO2
EG01_08G023720	18	1	8.4406E-13	0	6.0935E-05	senescence-specific cysteine protease SAG39-like
EG01_03G005730	18	2	6.7894E-13	647	6.0698E-05	zinc finger protein NUTCRACKER-like
EG01_01G009490	18	1	6.115E-13	0	6.0427E-05	Protein SAR DEFICIENT 1 OS%3DArabidopsis thaliana GN%3DSARD1 PE%3D1 SV%3D1
EG01_08G015180	18	1	6.115E-13	0	6.0427E-05	protein ASPARTIC PROTEASE IN GUARD CELL 2-like
EG01_04G019370	1	4	2.7498E-13	7673	5.8803E-05	12-oxophytodienoate reductase 2 OS%3DArabidopsis thaliana GN%3DOPR2 PE%3D1 SV%3D2
EG01_12G003400	18	2	2.6893E-13	1292	5.8679E-05	LOB domain-containing protein 1-like
EG01_04G027670	18	3	2.5234E-13	3219	5.8734E-05	short-chain dehydrogenase reductase 3b-like
EG01_12G014100	18	3	2.5206E-13	2580	5.8727E-05	Conserved hypothetical protein
EG01_05G006820	18	1	2.5069E-13	0	5.8665E-05	GDSL esterase lipase At5g33370-like RCOM_0610840
EG01_13G004080	18	1	2.5069E-13	0	5.8665E-05	CBS domain-containing protein CBSX5-like
EG01_U02G021280	18	1	2.5069E-13	0	5.8665E-05	thaumatin-like protein 1
EG01_09G008950	1	1	2.1709E-13	0	0.00005872	sphinganine C(4)-monooxygenase 2-like
EG01_06G003770	18	1	1.495E-13	0	5.8404E-05	Putative WRKY transcription factor 41 isoform X2
EG01_15G013120	1	4	9.8536E-14	3222	0.00005668	Putative WRKY transcription factor 67
EG01_U03G000360	1	2	8.5108E-14	2576	5.6673E-05	Putative LRR receptor-like serine
EG01_U03G012070	18	3	8.3795E-14	1937	5.6603E-05	glucan endo-1%2C3-beta-glucosidase-like
EG01_09G003690	18	2	8.2822E-14	647	5.6539E-05	Conserved hypothetical protein
EG01_01G000320	1	1	7.9304E-14	0	5.6648E-05	UDP-glycosyltransferase 73C6-like
EG01_10G005490	18	2	7.7627E-14	647	5.6583E-05	protein kinase PINOID-like
EG01_U02G010240	18	2	7.7627E-14	647	5.6583E-05	cinnamoyl-CoA reductase 1-like
EG01_09G004400	18	1	7.2779E-14	0	5.6583E-05	hepatoma-derived growth factor-related protein 2 isoform X1
EG01_12G007660	1	2	2.3274E-14	1292	5.4687E-05	WAT1-related protein At5g07050-like
EG01_03G000840	1	1	2.1693E-14	0	5.4675E-05	Protein phosphatase 2C 35 OS%3DOryza sativa subsp. japonica GN%3DXB15 PE%3D1 SV%3D1
EG01_12G013870	1	1	2.1693E-14	0	5.4675E-05	expansin-A2-like isoform X1
EG01_08G018430	1	2	2.0118E-14	1935	5.4687E-05	auxin-induced protein 22D
EG01_04G028150	18	2	1.97E-14	647	5.4609E-05	amino acid permease 6-like
EG01_10G009790	18	1	1.8445E-14	0	5.4603E-05	aspartic proteinase Asp1-like
EG01_12G008240	18	1	1.8241E-14	0	5.4543E-05	NADPH:quinone oxidoreductase-like
EG01_U02G031740	18	1	1.7089E-14	0	5.4585E-05	isoflavone reductase-like protein
EG01_U03G002730	18	1	1.7089E-14	0	5.4585E-05	geraniol 8-hydroxylase-like isoform X2
EG01_08G017500	1	2	7.1781E-15	647	5.2823E-05	primary amine oxidase
EG01_U03G015390	1	2	6.2319E-15	1292	5.2829E-05	Conserved hypothetical protein
EG01_10G007350	18	1	5.6764E-15	0	5.2745E-05	Conserved hypothetical protein
EG01_U03G013040	1	1	1.5833E-15	0	5.1078E-05	homeobox-leucine zipper protein HOX18-like
EG01_04G013640	1	2	1.4815E-15	647	5.1088E-05	polygalacturonase inhibitor 1-like
EG01_U02G008500	1	1	4.3417E-16	0	4.9454E-05	E3 ubiquitin-protein ligase MBR1-like
