# Supplementary material for: The Dynamic Interaction between Oil Palm and Phytophthora palmivora in Bud Rot Disease: Insights from Transcriptomic Analysis and Network Modelling
Source: J Fungi (Basel). 2024 Feb 20;10(3):164. doi: 10.3390/jof10030164 (PMC10971723; doi:10.3390/jof10030164)
Supplement: Supplementary file 1 [file jof-10-00164-s001.zip › Supp JOF/Table S3.pdf]

Table S3. List of primers for qRT-PCR

Name	Sequence (5'->3')
ELI_26439_FW	CTACTCGATGCTGACGGCCA
ELI_26439_RW	TGATTACCAGGCCGCTCGTT
CBM_20964-FW	GCTGCCAGAAGAAGAAGTCG
CBM_20964-RW	CTGGTTGGGTTCTTGCACTT
CBX_00541-FW	GCGTCATTTCACACGAGCTA
CBX_00541-RW	TCGAACGACTGCTTCATGTC
PPDK_40028-FW	GGTGGTGACCCACAGTCGAT
PPDK_40028-FW	GCACCGGGATTCTACCCCTC
GH17_FW	GGAGGCGTACTTCGGTCTGT
GH17_RW	GCCACCCACAACGGTGATTG
NEP1_47062_FW	TGCACTGGGTCTAGTCTTGG
NEP1_47062_RW	AAGACGCTGAGAGTGCCACA
RXLR_40906_FW	AATTCAGCTGGTGGGATGAC
RXLR_40906_RW	CGCCTTGTCATTCAAACCTT
HPM1_46444_FW	GGTCGTGCTAGTAGTTCGGG
HPM1_46444_RW	CTGTTTCGAGAGGACCCACC
