# Supplementary material for: The Dynamic Interaction between Oil Palm and Phytophthora palmivora in Bud Rot Disease: Insights from Transcriptomic Analysis and Network Modelling
Source: J Fungi (Basel). 2024 Feb 20;10(3):164. doi: 10.3390/jof10030164 (PMC10971723; doi:10.3390/jof10030164)
Supplement: Supplementary file 1 [file jof-10-00164-s001.zip › Supp JOF/Table S1.pdf]

Table S1. Gene hubs description of the co-expression network of *P. palmivora* Clon57 and Clon34

Gene	Module	Degree	Hub Score	Betweenness	Closeness	Annotation
PpalZC01_tig00034497_48158	2	11	1.0000000000	4545	3.07E-04	Elicitin
PpalZC01_tig00002349_32280	16	3	0.1999400000	4899	3.30E-04	hypothetical protein PC110_g23674, partial
PpalZC01_tig00000064_00541	2	3	0.1961900000	2064	2.85E-04	Para-nitrobenzyl esterase
PpalZC01_tig00002656_40028	16	3	0.1833800000	7655	3.56E-04	Pyruvate, phosphate dikinase 1
PpalZC01_tig00000179_02501	2	1	0.1543500000	0	2.84E-04	Ferric reductase
PpalZC01_tig00000448_05891	2	1	0.1543500000	0	2.84E-04	Alpha/Beta hydrolase fold
PpalZC01_tig00000909_12080	2	1	0.1543500000	0	2.84E-04	Amino acid/auxin permease-like protein
PpalZC01_tig00001069_13773	2	1	0.1543500000	0	2.84E-04	Mannan polymerase complex subunit mnn9
PpalZC01_tig00002225_28756	2	1	0.1543500000	0	2.84E-04	ABC transporter B family member 2
PpalZC01_tig00002368_32904	2	1	0.1543500000	0	2.84E-04	transmembrane protein, putative
PpalZC01_tig00002652_39843	2	1	0.1543500000	0	2.84E-04	hypothetical protein PHPALM_28109
PpalZC01_tig00002832_43103	2	1	0.1543500000	0	2.84E-04	hypothetical protein PHMEG_00018346
PpalZC01_tig00000759_10036	2	4	0.1483800000	786	2.66E-04	Niemann-Pick C1 protein
PpalZC01_tig00000185_02656	2	2	0.1227200000	783	2.66E-04	NADH dehydrogenase complex I
PpalZC01_tig00002379_33378	16	1	0.1119900000	0	3.04E-04	Jacalin-like lectin domain
PpalZC01_tig00001255_15663	9	8	0.0938510000	18971	3.83E-04	Histone-lysine N-methyltransferase EZH2
PpalZC01_tig00000578_07589	9	3	0.0662060000	5804	3.54E-04	Glycosyl transferase
PpalZC01_tig00003107_47069	13	3	0.0661910000	16805	3.95E-04	Carbohydrate-binding protein
PpalZC01_tig00000783_10460	15	3	0.0660280000	6425	3.55E-04	Hypothetical protein PHPALM_9685
PpalZC01_tig00034537_48667	9	3	0.0635600000	525	3.49E-04	Hypothetical protein PHPALM_18169
PpalZC01_tig00002332_31988	9	2	0.0575450000	263	3.49E-04	Glutathione S-transferase
PpalZC01_tig00002875_43886	9	1	0.0525700000	0	3.48E-04	hypothetical protein PHYSODRAFT_502568
PpalZC01_tig00002069_23912	9	1	0.0525700000	0	3.48E-04	Diacylglycerol O-acyltransferase 2
PpalZC01_tig00002002_21739	16	6	0.0342290000	2325	3.03E-04	Dual specificity protein phosphatase
PpalZC01_tig00002410_33925	16	2	0.0335870000	2540	3.28E-04	hypothetical protein PHYSODRAFT_502568
PpalZC01_tig00001104_14050	2	1	0.0229020000	0	2.49E-04	Hypothetical protein PHPALM_3592
PpalZC01_tig00002073_24101	2	1	0.0229020000	0	2.49E-04	voltage-gated potassium channel subunit beta
PpalZC01_tig00002190_27936	2	1	0.0229020000	0	2.49E-04	Glyoxylate/hydroxypyruvate reductase A
PpalZC01_tig00003032_46444	2	3	0.0229020000	525	2.49E-04	haustorium-specific membrane protein, putative
PpalZC01_tig00001102_14012	15	3	0.0138360000	6111	3.30E-04	2-hydroxyacid dehydrogenase
PpalZC01_tig00002615_39219	9	3	0.0131580000	5123	3.29E-04	Cationic amino acid transporter, C-terminal
PpalZC01_tig00002309_31260	13	3	0.0128960000	16883	4.06E-04	---NA---
PpalZC01_tig00001959_20000	2	1	0.0128290000	0	2.33E-04	ABC transporter G family member 31
PpalZC01_tig00002225_28599	2	1	0.0128290000	0	2.33E-04	Ferric reduction oxidase 7
PpalZC01_tig00002680_40471	15	5	0.0123420000	1816	3.05E-04	Lysosomal thioesterase PPT2
PpalZC01_tig00002710_41079	3	4	0.0116720000	4745	3.06E-04	Acyl-CoA desaturase
PpalZC01_tig00000550_07266	13	2	0.0114220000	1040	3.59E-04	Thaumatin protein
PpalZC01_tig00002500_36452	15	4	0.0112690000	3997	3.07E-04	putative aldehyde dehydrogenase
PpalZC01_tig00002094_24939	9	2	0.0111860000	263	3.24E-04	NAD-specific glutamate dehydrogenase
PpalZC01_tig00000555_07315	15	1	0.0101910000	0	3.25E-04	Diacylglycerol O-acyltransferase 2
PpalZC01_tig00000207_03002	9	1	0.0098104000	0	3.19E-04	Mannitol dehydrogenase
PpalZC01_tig00000547_07214	9	1	0.0098104000	0	3.19E-04	NAD(P)H:quinone oxidoreductase, type IV
PpalZC01_tig00002225_28864	13	3	0.0093807000	18815	4.15E-04	Carbohydrate esterase
PpalZC01_tig00002623_39425	9	1	0.0088819000	0	3.19E-04	ATP-binding Cassette (ABC) Superfamily
PpalZC01_tig00002583_38383	13	2	0.0079786000	524	3.67E-04	Zinc ion binding protein
PpalZC01_tig00001406_17014	13	3	0.0078131000	785	3.29E-04	Carbohydrate-binding protein
PpalZC01_tig00000378_05177	9	1	0.0073703000	0	3.02E-04	Glutathione S-transferase
PpalZC01_tig00002699_40906	9	1	0.0062656000	0	2.99E-04	Secreted RxLR effector peptide protein
PpalZC01_tig00002296_30720	16	2	0.0058354000	524	2.81E-04	ABC transporter B family member 11
PpalZC01_tig00002620_39343	16	2	0.0058354000	524	2.81E-04	Hypothetical protein PHPALM_15211
PpalZC01_tig00001978_20964	16	1	0.0052831000	0	2.81E-04	Cellulose-binding domain, fungal
PpalZC01_tig00003021_46192	16	1	0.0052831000	0	2.81E-04	Ricin-type beta-trefoil lectin domain
PpalZC01_tig00002735_41549	16	1	0.0052831000	0	2.81E-04	AGC/PKA protein kinase
PpalZC01_tig00000597_08013	3	7	0.0038357000	2823	2.85E-04	Gamma carbonic anhydrase 1
PpalZC01_tig00034577_49052	16	2	0.0035780000	263	2.62E-04	cell 5A endo-1,4-betaglucanase
PpalZC01_tig00003124_47251	16	2	0.0035780000	263	2.62E-04	Annexin protein
PpalZC01_tig00000373_05083	4	5	0.0028037000	3296	2.86E-04	Glutamate decarboxylase
PpalZC01_tig00002234_29022	3	2	0.0024361000	1295	2.66E-04	Papain-like cysteine protease C1
PpalZC01_tig00002285_30220	15	3	0.0023033000	525	2.83E-04	CAMK/CAMKL protein Kinase
PpalZC01_tig00002911_44455	4	4	0.0021843000	2310	2.67E-04	Beta-glucan synthesis-associated protein SKN1
PpalZC01_tig00000064_00565	3	1	0.0021485000	0	2.65E-04	Dicarboxylate/amino acid:cation (Na or H) symporter (DAACS) family protein
PpalZC01_tig00000675_08759	3	1	0.0021485000	0	2.65E-04	Dicarboxylate/amino acid:cation (Na or H) symporter (DAACS) family protein
PpalZC01_tig00000935_12308	3	1	0.0021485000	0	2.65E-04	NAD-specific glutamate dehydrogenase
PpalZC01_tig00001406_17013	3	1	0.0021485000	0	2.65E-04	elicitin-like protein
PpalZC01_tig00003107_47070	3	1	0.0021485000	0	2.65E-04	elicitin-like protein
PpalZC01_tig00002425_34342	15	2	0.0020853000	263	2.82E-04	Calcineurin-like phosphoesterase
PpalZC01_tig00002864_43702	3	2	0.0020427000	1295	2.84E-04	hypothetical protein GQ600_23012
PpalZC01_tig00003107_47068	1	4	0.0020316000	3559	3.78E-04	Necrosis inducing-like protein NPP1 type
PpalZC01_tig00002069_23935	15	1	0.0019050000	0	2.82E-04	MICAL-like protein
PpalZC01_tig00002262_29746	15	1	0.0019050000	0	2.82E-04	Uracil phosphoribosyltransferase
PpalZC01_tig00002069_23622	13	3	0.0018195000	17975	4.19E-04	salicylate hydroxylase, putative
PpalZC01_tig00002425_34337	3	1	0.0018016000	0	2.83E-04	Gamma carbonic anhydrase 1
PpalZC01_tig00000723_09435	15	1	0.0017393000	0	2.84E-04	DNA excision repair protein ERCC-6
PpalZC01_tig00034497_48200	15	1	0.0017393000	0	2.84E-04	NADH dehydrogenase complex I
PpalZC01_tig00000242_03279	4	1	0.0015704000	0	2.66E-04	Kazal-type serine protease inhibitor domain
PpalZC01_tig00000332_04320	4	1	0.0015704000	0	2.66E-04	Protein-tyrosine phosphatase-like
PpalZC01_tig00002652_39853	4	1	0.0015704000	0	2.66E-04	EF-hand domain pair
PpalZC01_tig00001959_20009	3	4	0.0015621000	1045	2.65E-04	mannitol dehydrogenase, putative
PpalZC01_tig00002509_36566	13	2	0.0013480000	263	3.35E-04	L-asparaginase 1

PpalZC01_tig00002013_22128	13	2	0.0013201000	263	3.03E-04	Alkaline phosphatase
PpalZC01_tig00002130_26101	15	1	0.0012902000	0	2.63E-04	hypothetical protein PHPALM_31901, partial
PpalZC01_tig00002566_38148	15	1	0.0012902000	0	2.63E-04	hypothetical protein PHPALM_31901, partial
PpalZC01_tig00001055_13615	1	2	0.0012765000	1548	3.45E-04	putative RxLR effector
PpalZC01_tig00002541_37462	13	3	0.0012680000	17660	4.22E-04	Beta-elicitin DRE-beta
PpalZC01_tig00000019_00050	1	2	0.0012596000	1040	3.45E-04	Hypothetical protein PHPALM_15314
PpalZC01_tig00003062_46728	1	2	0.0012457000	263	3.44E-04	Transglutaminase elicitor
PpalZC01_tig00002625_39477	13	1	0.0012059000	0	3.02E-04	ATP-binding Cassette (ABC) Superfamily
PpalZC01_tig00002357_32609	15	1	0.0011681000	0	2.63E-04	secreted RxLR effector peptide protein, putative
PpalZC01_tig00002907_44305	13	2	0.0011395000	1040	3.79E-04	Phenol acid carboxylase
PpalZC01_tig00000476_06311	13	1	0.0007550900	0	3.08E-04	Acyl-CoA desaturase
PpalZC01_tig00002310_31448	13	1	0.0007394200	0	2.80E-04	putative auto-transporter adhesin head GIN domain
PpalZC01_tig00000568_07446	16	1	0.0005522500	0	2.45E-04	Formate dehydrogenase
PpalZC01_tig00002049_23278	16	1	0.0005522500	0	2.45E-04	Ventral vesicle protein
PpalZC01_tig00002258_29679	3	4	0.0005133500	1045	2.49E-04	GPI-anchored leucine-rich lipoprotein
PpalZC01_tig00000064_00651	4	3	0.0004215300	1556	2.50E-04	secreted RxLR effector peptide protein, putative
PpalZC01_tig00000807_10707	4	1	0.0003371300	0	2.50E-04	Reverse transcriptase (RNA-dependent DNA polymerase)
PpalZC01_tig00002062_23517	4	1	0.0003371300	0	2.50E-04	Mitochondrial phosphate carrier protein 3
PpalZC01_tig00002494_36297	3	2	0.0003147600	263	2.34E-04	RxLR effector family
PpalZC01_tig00000361_04643	4	3	0.0002883300	785	2.35E-04	cell 5A endo-1,4-betaglucanase
PpalZC01_tig00001164_14903	3	1	0.0002875500	0	2.34E-04	ethanolamine kinase A
PpalZC01_tig00002093_24930	3	1	0.0002875500	0	2.34E-04	NAD-specific glutamate dehydrogenase
PpalZC01_tig00002069_23878	3	2	0.0002639300	263	2.48E-04	NAD(P)H:quinone oxidoreductase, type IV
PpalZC01_tig00002842_43356	4	2	0.0002584600	263	2.35E-04	YrhK-like protein
PpalZC01_tig00002139_26433	1	3	0.0002472600	1299	3.18E-04	highly acidic elicitin
PpalZC01_tig00000176_02258	3	1	0.0002411100	0	2.48E-04	Glutamate dehydrogenase 2
PpalZC01_tig00002735_41585	3	1	0.0002411100	0	2.48E-04	Calcium-transporting ATPase 1
PpalZC01_tig00002541_37456	13	2	0.0002300100	17415	4.23E-04	highly acidic elicitin 20
PpalZC01_tig00000597_08016	7	5	0.0002222200	19577	4.24E-04	Calcineurin-like phosphoesterase
PpalZC01_tig00002859_43636	1	2	0.0002171100	783	3.17E-04	Phenol acid carboxylase
PpalZC01_tig00001968_20507	13	3	0.0002147700	785	3.45E-04	folate-Biopterin Transporter (FBT) family
PpalZC01_tig00002541_37455	13	2	0.0002142400	263	3.80E-04	acidic elicitin
PpalZC01_tig00002828_42996	1	1	0.0001922700	0	3.16E-04	Bidirectional sugar transporter SWEET11
PpalZC01_tig00002378_33307	1	4	0.0001870100	786	2.94E-04	Small secreted protein
PpalZC01_tig00000207_02999	3	1	0.0001478400	0	2.33E-04	ABC transporter G family member 31
PpalZC01_tig00002069_23623	1	3	0.0001470400	525	2.93E-04	putative RxLR effector
PpalZC01_tig00002541_37460	1	1	0.0001385000	0	2.93E-04	acidic elicitin
PpalZC01_tig00003030_46304	13	2	0.0001316900	263	3.17E-04	Inorganic phosphate transporter
PpalZC01_tig00001961_20178	13	1	0.0001203000	0	3.17E-04	Hypothetical protein PHPALM_15314
PpalZC01_tig00002680_40470	13	1	0.0001200000	0	3.45E-04	RxLR effector protein
PpalZC01_tig00002541_37459	7	4	0.0000490150	3065	3.85E-04	highly acidic elicitin 20
PpalZC01_tig00000137_01668	4	2	0.0000487150	263	2.22E-04	Plasma-membrane proton-efflux P-type ATPase
PpalZC01_tig00002139_26438	3	1	0.0000485830	0	2.20E-04	highly acidic elicitin
PpalZC01_tig00000395_05447	8	3	0.0000454750	17471	4.18E-04	Phosphatidate cytidylyltransferase
PpalZC01_tig00001438_17321	4	1	0.0000445040	0	2.22E-04	Tartrate-resistant acid phosphatase type 5
PpalZC01_tig00001957_19869	8	5	0.0000416050	18384	4.11E-04	Carbohydrate-binding protein
PpalZC01_tig00003030_46348	4	1	0.0000398930	0	2.21E-04	Glutathione transferase, theta class
PpalZC01_tig00003030_46305	7	2	0.0000379210	783	3.82E-04	Sugar (and other) transporter
PpalZC01_tig00000345_04433	7	4	0.0000375280	1302	3.51E-04	necrosis inducing-like protein NPP1 type
PpalZC01_tig00002351_32382	7	1	0.0000342990	0	3.81E-04	Hypothetical protein PHPALM_10086
PpalZC01_tig00002139_26432	8	3	0.0000307980	525	3.77E-04	acidic elicitin
PpalZC01_tig00002102_25101	7	2	0.0000303570	1040	3.51E-04	Glucan 1,3-beta-glucosidase
PpalZC01_tig00002425_34375	1	1	0.0000288640	0	2.73E-04	Carbohydrate-binding protein
PpalZC01_tig00002562_37971	1	1	0.0000288640	0	2.73E-04	NmrA-like family protein, putative
PpalZC01_tig00002735_41606	1	1	0.0000288640	0	2.73E-04	beta-glucosidase btgE
PpalZC01_tig00000186_02694	7	1	0.0000274550	0	3.50E-04	Beta-elicitin DRE-beta
PpalZC01_tig00000161_02094	4	1	0.0000272880	0	2.09E-04	WRKY transcription factor 19
PpalZC01_tig00002069_23687	7	2	0.0000234610	524	3.48E-04	Cortactin-binding protein 2
PpalZC01_tig00000021_00093	1	1	0.0000226950	0	2.72E-04	Pleiotropic drug resistance protein 3
PpalZC01_tig00002783_42396	1	1	0.0000226950	0	2.72E-04	Secreted RxLR effector peptide protein
PpalZC01_tig00000578_07590	13	1	0.0000203250	0	2.92E-04	Carbohydrate-binding protein
PpalZC01_tig00002763_42118	8	3	0.0000077641	525	3.71E-04	Serine/threonine protein Kinase
PpalZC01_tig00002182_27565	14	2	0.0000073999	16128	3.99E-04	Carbohydrate-binding protein
PpalZC01_tig00001536_17892	8	2	0.0000072153	2295	3.73E-04	Amino Acid/Auxin Permease (AAAP) Family
PpalZC01_tig00002562_37970	8	1	0.0000064216	0	3.71E-04	FYVE zinc finger domain-containing protein
PpalZC01_tig00002828_42991	7	2	0.0000063979	524	3.22E-04	Histidine phosphatase superfamily (branch 2)
PpalZC01_tig00002214_28258	14	4	0.0000063379	18023	3.88E-04	Histidine phosphatase superfamily (branch 2)
PpalZC01_tig00000727_09544	7	1	0.0000057924	0	3.21E-04	Acetyl-coenzyme A synthetase
PpalZC01_tig00002296_30711	7	1	0.0000057924	0	3.21E-04	Phosphoethanolamine
PpalZC01_tig00001406_17027	7	2	0.0000051802	783	3.22E-04	Necrosis and ethylene-inducing protein 6
PpalZC01_tig00003037_46526	8	3	0.0000051420	2060	3.42E-04	Major Facilitator Superfamily (MFS)
PpalZC01_tig00002069_23944	8	1	0.0000047535	0	3.43E-04	putative glycosyl hydrolase family 30 protein
PpalZC01_tig00002139_26441	8	1	0.0000047535	0	3.43E-04	Beta-elicitin DRE-beta
PpalZC01_tig00000305_04020	8	1	0.0000043490	0	3.38E-04	Cell 12A endoglucanase
PpalZC01_tig00034559_48903	8	1	0.0000043490	0	3.38E-04	Glycerophosphoryl diester phosphodiesterase
PpalZC01_tig00002288_30397	7	2	0.0000039639	263	3.19E-04	P-loop containing nucleoside triphosphate hydrolase
PpalZC01_tig00002013_22121	7	2	0.0000039229	263	2.97E-04	NAD(P)-binding domain
PpalZC01_tig00002310_31482	7	2	0.0000032050	524	2.97E-04	NmrA-like family protein, putative
PpalZC01_tig00000242_03280	7	1	0.0000022204	0	2.94E-04	Kazal-type serine protease inhibitor domain
PpalZC01_tig00002179_27376	11	7	0.0000019437	2823	3.30E-04	Cell 12A endoglucanase
PpalZC01_tig00002196_28021	14	4	0.0000016298	6401	3.59E-04	Annexin protein
PpalZC01_tig00001245_15612	12	6	0.0000015293	13854	3.51E-04	secreted RxLR effector peptide protein, putative
PpalZC01_tig00000695_08962	14	4	0.0000012674	2061	3.29E-04	Sugar transporter
PpalZC01_tig00002772_42258	12	2	0.0000012143	12935	3.69E-04	Sulfate Permease

PpalZC01_tig00000361_04663	8	4	0.0000010958	1303	3.15E-04	Sulfate Permease (SulP) Family
PpalZC01_tig00003107_47096	14	2	0.0000010708	263	3.52E-04	ABC transporter G family member 15
PpalZC01_tig00002437_34939	14	2	0.0000010105	1040	3.29E-04	Formate dehydrogenase
PpalZC01_tig00001968_20532	8	2	0.0000008688	263	3.14E-04	Avr1b-1 Avirulence-like protein
PpalZC01_tig00000207_02992	8	2	0.0000006719	263	2.91E-04	mannitol dehydrogenase, putative
PpalZC01_tig00002205_28108	8	2	0.0000006719	263	2.91E-04	Elicitin
PpalZC01_tig00002988_45672	8	1	0.0000006138	0	2.91E-04	Purine-cytosine permease
PpalZC01_tig00000164_02111	7	1	0.0000006055	0	2.75E-04	Glycosyl hydrolases family 6
PpalZC01_tig00002180_27475	14	1	0.0000005998	0	3.22E-04	Mannitol dehydrogenase
PpalZC01_tig00000486_06422	7	2	0.0000005415	263	2.75E-04	Magnesium-dependent phosphatase-1
PpalZC01_tig00000255_03536	8	1	0.0000004866	0	2.90E-04	secreted RxLR effector peptide protein, putative
PpalZC01_tig00003035_46495	12	6	0.0000004433	9289	3.30E-04	Glutathione transferase, theta class
PpalZC01_tig00000751_09861	10	7	0.0000003922	1563	3.01E-04	Short chain dehydrogenase
PpalZC01_tig00000277_03780	11	2	0.0000003402	1295	3.05E-04	Amino acid/polyamine transporter family 1
PpalZC01_tig00000582_07724	10	3	0.0000003370	4031	3.25E-04	Adenine phosphoribosyltransferase
PpalZC01_tig00002102_25153	7	1	0.0000003033	0	2.57E-04	Cell 12A endoglucanase
PpalZC01_tig00000909_12086	11	1	0.0000003000	0	3.04E-04	Amino Acid/Auxin Permease (AAAP) Family
PpalZC01_tig00001970_20615	11	1	0.0000003000	0	3.04E-04	RxLR effector protein
PpalZC01_tig00000500_06538	11	1	0.0000003000	0	3.04E-04	Lysosomal thioesterase PPT2-B
PpalZC01_tig00000635_08440	11	1	0.0000003000	0	3.04E-04	Major intrinsic protein
PpalZC01_tig00003107_47071	11	1	0.0000003000	0	3.04E-04	Acidic elicitin A1
PpalZC01_tig00001580_18261	6	2	0.0000002881	6815	3.09E-04	Sugar (and other) transporter
PpalZC01_tig00002139_26440	12	2	0.0000002861	1295	3.05E-04	elicitin-like protein 6 precursor
PpalZC01_tig00002562_38096	12	2	0.0000002718	263	3.04E-04	Peroxiredoxin-2
PpalZC01_tig00001591_18312	12	2	0.0000002636	783	3.22E-04	Trans-aconitate 2-methyltransferase
PpalZC01_tig00002575_38317	10	4	0.0000002617	2061	3.01E-04	Retrotransposon gag protein
PpalZC01_tig00001438_17322	11	4	0.0000002601	1045	2.83E-04	Tartrate-resistant acid phosphatase type 5
PpalZC01_tig00000854_11354	12	1	0.0000002483	0	3.04E-04	hypothetical protein PHMEG_00016594
PpalZC01_tig00002225_28863	12	1	0.0000002483	0	3.04E-04	Glutamine amidotransferase class-I
PpalZC01_tig00002783_42376	14	3	0.0000002415	1301	3.04E-04	Zinc finger protein 76
PpalZC01_tig00001222_15319	12	1	0.0000002361	0	3.22E-04	putative alpha-L-arabinofuranosidase B
PpalZC01_tig00001968_20506	12	1	0.0000002361	0	3.22E-04	Glyceraldehyde-3-phosphate dehydrogenase
PpalZC01_tig00001551_17984	14	1	0.0000001956	0	3.03E-04	putative voltage-gated potassium channel subunit beta
PpalZC01_tig00002288_30394	14	1	0.0000001956	0	3.03E-04	Multidrug resistance protein ABC Superfamily
PpalZC01_tig00003030_46365	12	3	0.0000001785	525	2.98E-04	glutathione theta class
PpalZC01_tig00002509_36573	14	2	0.0000001742	783	3.03E-04	Secreted RxLR effector peptide protein
PpalZC01_tig00002073_24102	14	2	0.0000001494	524	2.82E-04	Hypothetical protein PHPALM_8560
PpalZC01_tig00002988_45620	14	2	0.0000001481	263	2.82E-04	Tartrate-resistant acid phosphatase type 5
PpalZC01_tig00000543_07095	14	3	0.0000001180	525	2.81E-04	Serine/threonine protein Kinase
PpalZC01_tig00000547_07218	8	1	0.0000001037	0	2.70E-04	NAD(P)H:quinone oxidoreductase, type IV
PpalZC01_tig00002139_26439	8	1	0.0000001037	0	2.70E-04	acidic elicitin
PpalZC01_tig00002929_44719	6	5	0.0000000710	6802	2.91E-04	putative RXLR effector PEXRD49_186_2
PpalZC01_tig00002179_27211	12	5	0.0000000675	1046	2.83E-04	Transglutaminase elicitor
PpalZC01_tig00002116_25535	10	1	0.0000000605	0	2.79E-04	Glycoside hydrolase catalytic N-terminal domain-containing protein
PpalZC01_tig00000207_03004	10	1	0.0000000605	0	2.79E-04	mannitol dehydrogenase, putative
PpalZC01_tig00002015_22246	10	1	0.0000000605	0	2.79E-04	Serine protease
PpalZC01_tig00002435_34854	10	1	0.0000000605	0	2.79E-04	M96 mating-specific protein family
PpalZC01_tig00002842_43325	10	1	0.0000000605	0	2.79E-04	Arabinogalactan endo-1,4-beta-galactosidase
PpalZC01_tig00003032_46413	10	1	0.0000000605	0	2.79E-04	Major Facilitator Superfamily (MFS)
PpalZC01_tig00000434_05732	10	3	0.0000000494	1299	2.80E-04	ABC transporter G family member 38
PpalZC01_tig00002310_31457	6	2	0.0000000444	4428	2.73E-04	NmrA-like family
PpalZC01_tig00001580_18263	6	2	0.0000000440	1040	2.71E-04	Sugar (and other) transporter
PpalZC01_tig00000373_05110	6	2	0.0000000440	524	2.70E-04	hypothetical protein PHPALM_419
PpalZC01_tig00001953_19631	11	2	0.0000000440	263	2.63E-04	Glycoside hydrolase catalytic N-terminal domain-containing protein
PpalZC01_tig00000828_11015	12	1	0.0000000420	0	2.82E-04	Cysteine-rich secretory protein family
PpalZC01_tig00002509_36626	10	1	0.0000000404	0	2.79E-04	Hypothetical protein PHPALM_13074
PpalZC01_tig00003107_47095	10	1	0.0000000404	0	2.79E-04	ABC transporter G family member 15
PpalZC01_tig00001968_20512	11	1	0.0000000402	0	2.63E-04	Cysteine-rich protein
PpalZC01_tig00002491_36101	11	1	0.0000000402	0	2.63E-04	putative exo-1,4-beta-xylosidase
PpalZC01_tig00000345_04434	6	1	0.0000000398	0	2.70E-04	Putative xylanase
PpalZC01_tig00002010_22009	12	1	0.0000000378	0	2.63E-04	voltage-gated potassium channel subunit beta
PpalZC01_tig00000324_04192	12	1	0.0000000378	0	2.63E-04	Glycoside hydrolase catalytic N-terminal domain-containing protein
PpalZC01_tig00002988_45621	12	1	0.0000000378	0	2.63E-04	Tartrate-resistant acid phosphatase type 5
PpalZC01_tig00003021_46235	12	1	0.0000000378	0	2.63E-04	Scavenger mRNA decapping enzyme C-term binding
PpalZC01_tig00000783_10459	10	2	0.0000000309	783	2.61E-04	Kazal-type serine protease inhibitor domain
PpalZC01_tig00002371_33034	10	1	0.0000000277	0	2.60E-04	WRKY transcription factor 19
PpalZC01_tig00001123_14265	12	1	0.0000000276	0	2.76E-04	Lytic transglycosylase
PpalZC01_tig00002710_41085	12	1	0.0000000276	0	2.76E-04	Adenosylhomocysteinase
PpalZC01_tig00001239_15517	14	2	0.0000000252	263	2.63E-04	Pectin methylesterase
PpalZC01_tig00001945_19254	11	1	0.0000000246	0	2.46E-04	Hypothetical protein PHPALM_18169
PpalZC01_tig00002623_39424	14	1	0.0000000229	0	2.62E-04	ABC transporter G family member 29
PpalZC01_tig00001055_13614	14	1	0.0000000182	0	2.62E-04	Salicylate hydroxylase
PpalZC01_tig00002188_27864	14	1	0.0000000182	0	2.62E-04	ribonuclease, putative
PpalZC01_tig00002447_35280	14	1	0.0000000141	0	2.46E-04	Niemann-Pick C1 protein
PpalZC01_tig00002317_31736	5	6	0.0000000087	4062	2.42E-04	NADH-ubiquinone reductase complex 1 MLRQ subunit
PpalZC01_tig00000142_01753	6	2	0.0000000082	4199	2.57E-04	Hypothetical protein PHPALM_15305
PpalZC01_tig00002307_31146	6	2	0.0000000076	783	2.53E-04	Alcohol dehydrogenase
PpalZC01_tig00003107_47062	6	2	0.0000000074	263	2.53E-04	Necrosis and ethylene-inducing protein 6
PpalZC01_tig00001968_20508	10	3	0.0000000058	525	2.44E-04	Histidine phosphatase superfamily
PpalZC01_tig00001434_17240	6	3	0.0000000051	525	2.37E-04	Putative Glycosyl hydrolase family 17 protein
PpalZC01_tig00002399_33783	6	1	0.0000000042	0	2.37E-04	Plasma membrane ATPase
PpalZC01_tig00000983_12796	10	1	0.0000000032	0	2.30E-04	Beta-glucan synthesis-associated protein SKN1
PpalZC01_tig00002988_45597	10	1	0.0000000032	0	2.30E-04	ABC transporter G family member 31
PpalZC01_tig00002865_43753	5	3	0.0000000016	785	2.28E-04	Mannan polymerase complex subunit mnn9

PpalZC01_tig00000568_07447	5	2	0.0000000015	1040	2.28E-04	Formate dehydrogenase
PpalZC01_tig00000783_10458	5	2	0.0000000015	1040	2.28E-04	Kazal-type serine protease inhibitor domain
PpalZC01_tig00002763_42109	5	1	0.0000000013	0	2.28E-04	Amino Acid/Auxin Permease (AAAP) Family
PpalZC01_tig00002807_42637	5	1	0.0000000013	0	2.28E-04	Pectin methylesterase
PpalZC01_tig00002509_36680	5	3	0.0000000010	785	2.16E-04	Glycoside hydrolase superfamily
PpalZC01_tig00002288_30399	5	3	0.0000000010	785	2.16E-04	Trans-aconitate 2-methyltransferase
PpalZC01_tig00000179_02289	5	2	0.0000000010	263	2.15E-04	Hypothetical protein PHPALM_4055
PpalZC01_tig00002288_30407	5	1	0.0000000009	0	2.15E-04	Trans-aconitate 2-methyltransferase
PpalZC01_tig00002180_27412	6	1	0.0000000008	0	2.24E-04	Pectinesterase
PpalZC01_tig00002538_37329	6	1	0.0000000008	0	2.24E-04	Secreted RxLR effector peptide protein
PpalZC01_tig00002309_31332	5	2	0.0000000002	263	2.04E-04	Sugar transporter
PpalZC01_tig00002763_42119	5	2	0.0000000002	263	2.04E-04	hypothetical protein PPTG_08953
PpalZC01_tig00000064_00696	5	1	0.0000000002	0	2.04E-04	glycoside hydrolase family 5 protein
PpalZC01_tig00000242_03281	5	1	0.0000000002	0	2.04E-04	hypothetical protein PHMEG_00031254
PpalZC01_tig00001123_14351	5	1	0.0000000002	0	2.04E-04	Hypothetical protein PHPALM_37279
PpalZC01_tig00003030_46303	5	1	0.0000000001	0	1.94E-04	Sugar (and other) transporter
PpalZC01_tig00002391_33698	5	1	0.0000000001	0	1.94E-04	Transmembrane protein
