# Supplementary figures and images for: The Dynamic Interaction between Oil Palm and Phytophthora palmivora in Bud Rot Disease: Insights from Transcriptomic Analysis and Network Modelling
Source: J Fungi (Basel). 2024 Feb 20;10(3):164. doi: 10.3390/jof10030164 (PMC10971723; doi:10.3390/jof10030164)

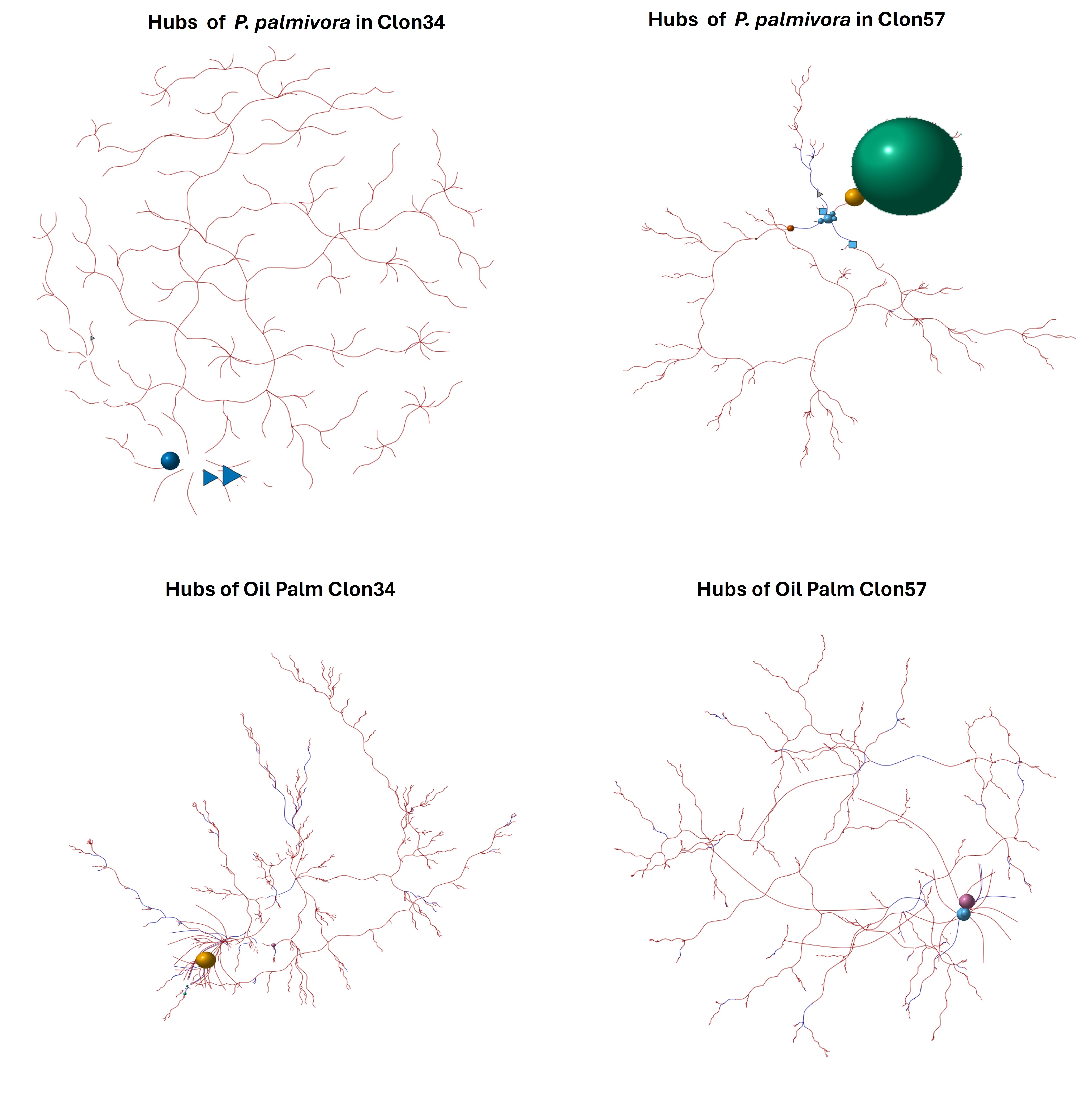

Supplement: Supplementary file 1 [file jof-10-00164-s001.zip › Supp JOF/Figure S3.jpg]

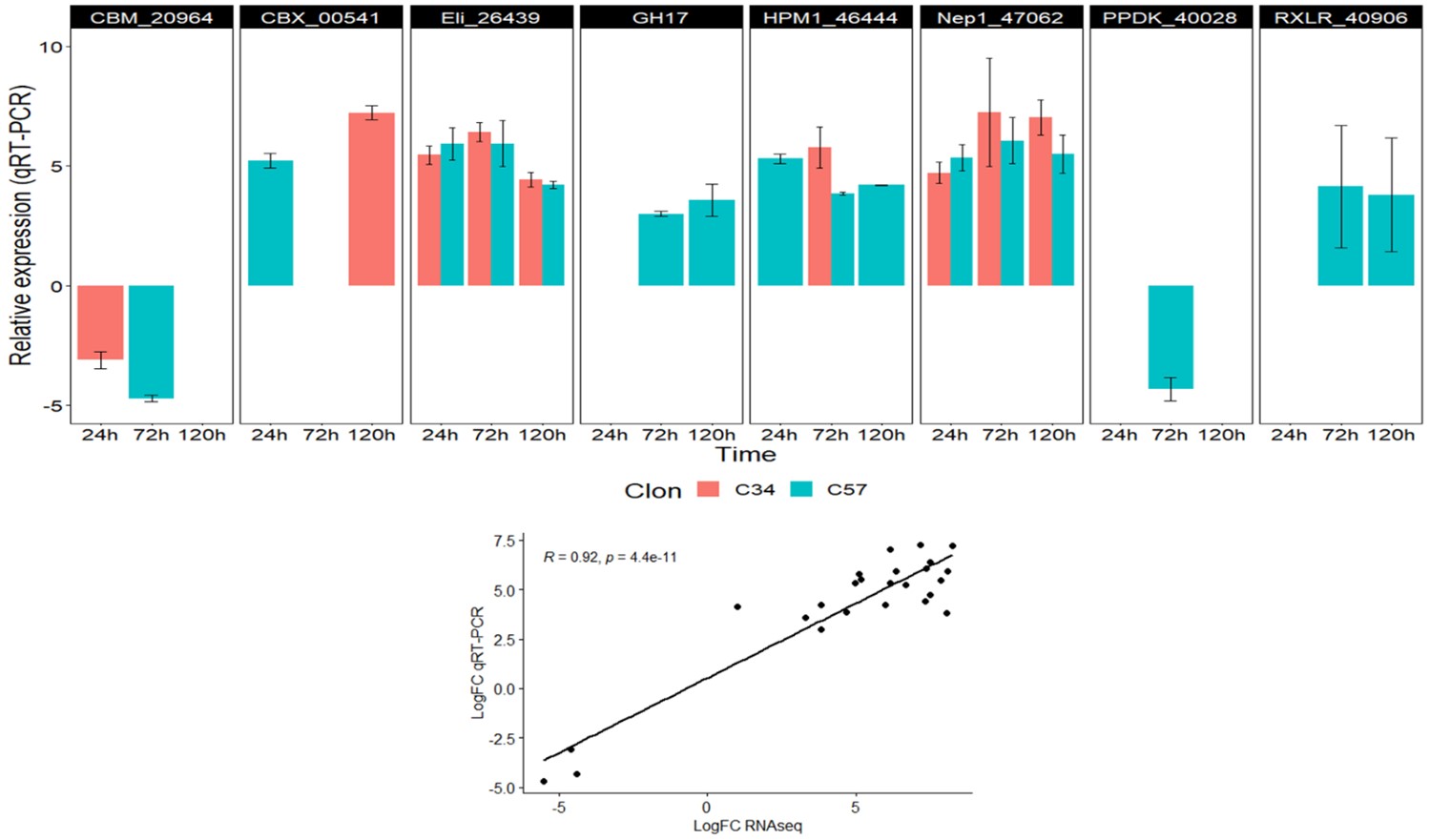

Supplement: Supplementary file 1 [file jof-10-00164-s001.zip › Supp JOF/Figure S2.jpg]

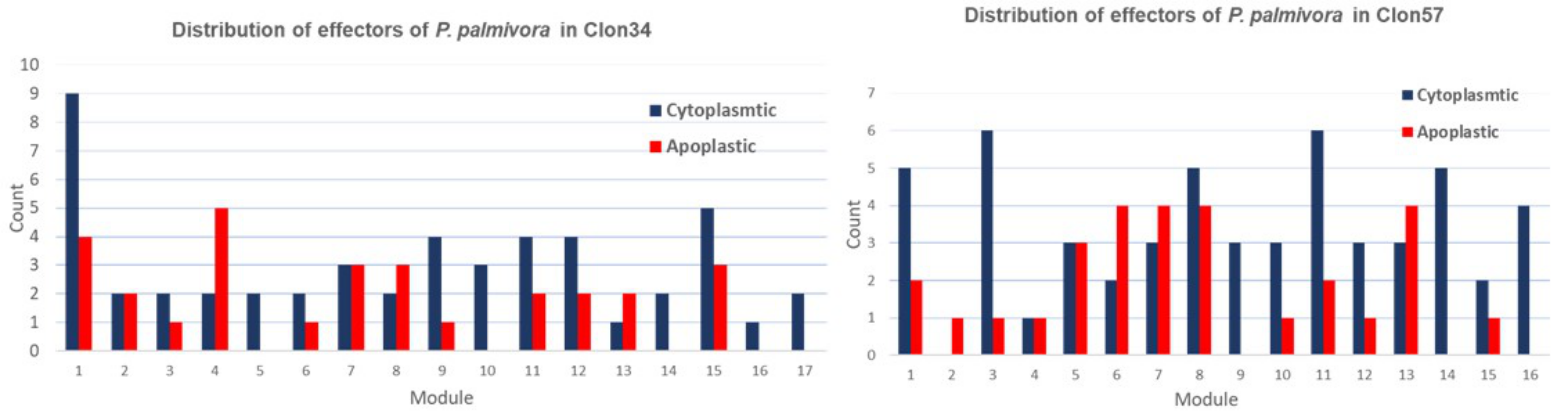

Supplement: Supplementary file 1 [file jof-10-00164-s001.zip › Supp JOF/Figure S1.png]
